# Acute Impact of Nonoptimal Ambient Temperatures on Plasma Levels of 3000 Proteins in Chinese Adults

**DOI:** 10.1021/acs.est.4c13020

**Published:** 2025-03-04

**Authors:** Yi Tong Guo, Mohsen Mazidi, Neil Wright, Pang Yao, Baihan Wang, Yue Niu, Xi Xia, Xia Meng, Cong Liu, Robert Clarke, Kin Bong Hubert Lam, Christiana Kartsonaki, Iona Millwood, Yiping Chen, Ling Yang, Huaidong Du, Canqing Yu, Dianjianyi Sun, Jun Lv, Liming Li, Junshi Chen, Maxim Barnard, Xiaocao Tian, Kin Fai Ho, Ka Hung Chan, Antonio Gasparrini, Haidong Kan, Zhengming Chen, the China Kadoorie Biobank Study Group

**Affiliations:** 1 JC School of Public Health and Primary Care, The Chinese University of Hong Kong, Hong Kong SAR, China; 2 Clinical Trial Service Unit and Epidemiological Studies Unit, Nuffield Department of Population Health, University of Oxford, Oxford OX3 7LF, U.K.; 3 School of Public Health, Key Lab of Public Health Safety of the Ministry of Education and NHC Key Lab of Health Technology Assessment, Fudan University, Shanghai 200433, China; 4 Department of Occupational and Environmental Health, School of Public Health, Xi’an Jiaotong University Health Science Center, Xi’an 710061, China; 5 Key Laboratory of Environment and Genes Related to Diseases, Ministry of Education, Xi’an 710000, China; 6 School of Public Health, Shaanxi University of Chinese Medicine, Xi’an 030001, China; 7 Department of Epidemiology and Biostatistics, School of Public Health, Peking University Health Science Center, Beijing 100871, China; 8 Peking University Center for Public Health and Epidemic Preparedness & Response, Beijing 100871, China; 9 Ministry of Education, Key Laboratory of Epidemiology of Major Diseases (Peking University),, Beijing 100071, China; 10 China National Center for Food Safety Risk Assessment, Beijing 100000, China; 11 Qingdao Center of Disease and Control and Prevention, Qingdao 266000, China; 12 Environment & Health Modelling (EHM) Lab, Department of Public Health Environments and Society, London School of Hygiene & Tropical Medicine, London WC1 E7H, U.K.; 13 Children’s Hospital of Fudan university, National Center for Children’s Health, Shanghai 200433, China

**Keywords:** temperature, short-term effects, proteomics, climate change, Chinese

## Abstract

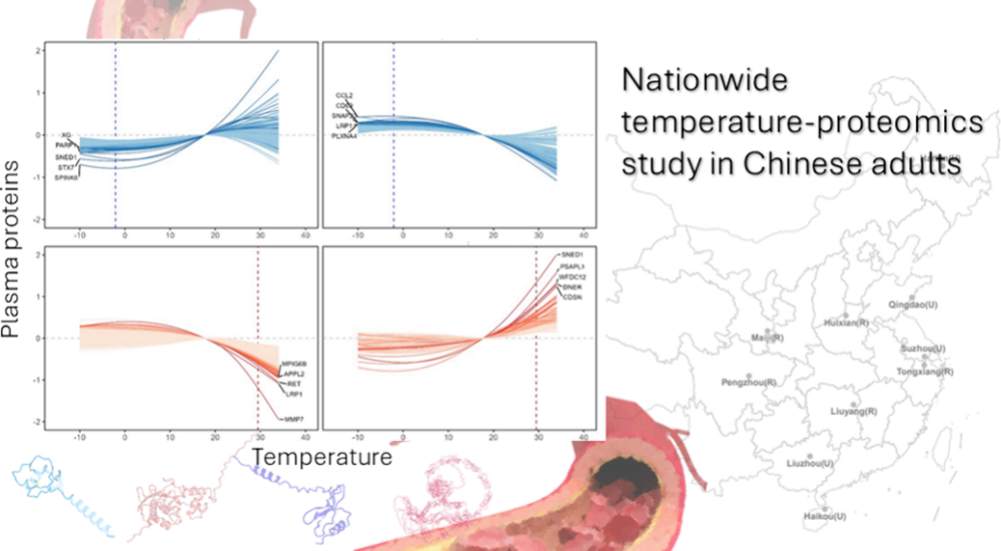

Nonoptimal ambient temperatures (i.e., cold and heat) are leading environmental determinants of major diseases worldwide, but the underlying pathological mechanisms are still poorly understood. We used distributed-lag nonlinear models to examine the associations of cold (5^th^ percentile: −2.1 °C) and heat (95^th^ percentile: 29.5 °C) with 2923 plasma proteins in 3926 adults from 10 areas across China. Overall, 949 proteins were significantly (5% false discovery rate) associated with ambient temperature, including 387 (216/171 down/upregulated) with cold, 770 (656/114 down/upregulated) with heat, and 208 with both cold and heat. Above the median reference temperature (17.7 °C), the associations were largely linear, while below it, they were nonlinear with attenuation below 5 °C, potentially reflecting mediation by heating. Among the 949 proteins, >80% were also associated with systolic blood pressure and incident ischemic heart disease risk and enriched in relevant pathological pathways (e.g., inflammation, immunity, and platelet aggregation). Our study provided a novel atlas of plasma proteins associated with nonoptimal temperatures in Chinese adults.

## Abstract 2

This study reveals significant associations between ambient temperatures and over 900 plasma proteins in the Chinese population, many of which correlate with increased systolic blood pressure and incident ischemic heart disease risks.

## Introduction

Climate change is considered as the “single biggest health threat facing humanity”.^1^ One mechanism by which climate change influences health is via higher ambient temperature and weather extremes,^2^ thereby altering population exposure to nonoptimal ambient temperatures (i.e., heat and cold), which have a worldwide relevance.^3^ Compared to the preindustrial level, the global surface temperature has increased by 1.1 °C in the past decade.^4^ Multinational ecological studies have identified varying optimal temperatures associated with minimal daily mortality across populations with different adaptation capacities, covering a general range of 18–25 °C.^5^ In 2019, nonoptimal ambient temperatures were estimated to account for >5 million deaths based on evidence derived from ecological studies on the short-term health impact.^5^

Despite accumulating evidence from ecological studies of nonoptimal temperatures on mortality,^5−10^ little is known about the biological mechanisms underlying these associations.^11^ Much of the available mechanistic evidence has focused on thermoregulation responses and a limited number of conventional physical traits (e.g., lung function, blood pressure [BP], and heart rate) or blood biomarkers (e.g., blood lipids and blood glucose).^12−18^ Similarly, although there have been relevant animal experiments or human physiological studies, most tended to focus on a few molecular mechanisms and were small and often restricted to young healthy adults with uncertain generalizability to real world settings, especially in low- and middle-income countries including China.^12−15,17^

Plasma proteins (e.g., interleukin-6 [IL6] and C-reactive protein [CRP]) are widely used biomarkers to predict disease risk.^19,20^ Accumulating evidence on large-scale proteomics assays in population-based biobanks has helped to clarify our understanding of the etiological roles of major risk factors (e.g., smoking and adiposity) and identified potential drug targets for several diseases.^21−25^ In recent years, a few studies of Western populations have also examined the associations of ambient temperature with the plasma levels of specific proteins. In a German study of ∼1100 older people, low temperature was associated with higher levels of 64 inflammation-related proteins, but the analyses were restricted to only 71 proteins and a relatively narrow temperature range (−7.8 to 24.7 °C).^26^ A recent study in ∼3000 US adults developed a composite “proteome score” based on 6347 proteins measured using the aptamer-based SomaScan assay as a proxy of long-term (5 year average) temperature exposures.^27^ However, such long-term average temperatures typically reflect the general neighborhood climate condition rather than day-to-day variations underlying the acute health effects of differences in ambient temperature. Moreover, the study findings in typical Western populations may not be readily generalizable to low- and middle-income countries, where few have adequate central heating or air conditioning. A more comprehensive investigation of the acute impact of temperature on the plasma proteome is required to improve our understanding of the mechanisms underlying the health impact of ambient temperature, and to discover the relevance of temperature on disease markers or therapeutic targets using state-of-the-art analytical methods in proteomic epidemiology.

We present detailed analyses of exposure–lag relationships between daily ambient temperatures with 2923 unique proteins measured using the Olink Explore 3072 platform among 3926 Chinese adults recruited from 10 areas in the prospective China Kadoorie Biobank (CKB). The present study aims to (1) discover protein markers that are associated with cold or heat (nonoptimal temperatures), (2) investigate the impact of individual-level adaptation factors on these associations, and (3) explore for biological mechanisms linking nonoptimal temperatures to cardiovascular disease (CVD).

## Materials and Methods

### Study Design and Population

Details of the study design and characteristics of CKB participants have been described previously.^28^ In 2004–2008, CKB surveyed ∼512,000 adults aged 30–79 years across 10 geographically diverse areas (Figure S1). At baseline, trained health workers administered a laptop-based questionnaire including sociodemographic, lifestyle, and environmental factors and medical history and recorded physical measurements (e.g., anthropometry and BP). All participants had a 10 mL nonfasting (with time since the last meal recorded as fasting time) blood sample collected, processed, and stored in liquid nitrogen. The present study involved a case-cohort subset of 1951 cases of incident ischemic heart disease (IHD) and 2026 randomly selected subcohort participants who had no prior history of cardiovascular disease at baseline.^21,29^

### Meteorological Data

We obtained data on daily mean air temperature (°C) and relative humidity (RH, %) from the widely used fifth-generation European Centre for Medium Range Weather Forecasts (ECMWF) reanalysis database for global climate and weather (ERA5) at a 0.1 × 0.1° spatial resolution.^30^ For each participant, we ascertained their geolocation using the address of the baseline survey clinics, which were set up to recruit participants living within ∼1 km radius of the clinics.^28^ Using the clinic geolocation for all participants, we extracted daily meteorological metrics from the ERA5 for 21 consecutive days prior to the date of blood sample collection at baseline (i.e., a total of 22 days) to assess a “time-lagged” association, consistent with the best practice in previous population studies of the impact of temperature on cause-specific mortality.^5^

### Proteomics Assay

Details of the Olink Explore assay and quality control (QC) measures in the CKB have been described elsewhere.^31,32^ The baseline plasma samples of the 3977 participants were retrieved from liquid nitrogen, thawed, and aliquoted into 96-well plates, including eight wells per plate for external QC samples (to determine the limit of detection). The plasma samples were then couriered to Olink laboratories in Uppsala, Sweden, and Boston, US, for proteomic profiling using the Olink Explore 3072 platform that included 2923 unique proteins in four panels across two batches (first batch: 1472 proteins in Sweden; second batch: 1469 proteins in the US). The results of proteomics assays were quantified in arbitrary Normalized Protein eXpression (NPX) units on a log2 scale. Six proteins were replicated across all four panels and showed high levels of consistency (*r* > 0.8), so only one measure for each duplicated protein was used in the analyses. NPX values were first adjusted for plate identifier numbers using linear regression models (to control for batch effects) and standardized by dividing the corresponding standard errors for subsequent analyses.

### Statistical Analysis

The primary analysis excluded individuals with missing data on temperature (*n* = 51) due to ambiguity of the baseline survey clinic location, leaving 3926 for the main analyses, including 2006 randomly selected subcohort participants.

We examined the distribution of baseline characteristics by tertiles of mean ambient temperature on the day of sample collection (day 0) and selected baseline characteristics (for subgroup analyses). In assessing the exposure–lag–response relationships of ambient temperature with the levels of 2923 proteins, we fitted Gaussian generalized additive models (GAMs)^33^ with distributed-lag nonlinear models (DLNMs),^34^ which allow for bidimensional assessment of potentially nonlinear and delayed associations between temperature and proteins. All analyses were adjusted for age, age^2^, sex, study areas, fasting time, fasting time^2^, year of blood collection, hour of blood collection in a day, time to blood processing, same-day mean RH, and case ascertainment status (for the whole case-cohort only).

Specifically, we used natural cubic splines with two knots equally spaced on the temperature distribution and quadratic B-splines with two knots equally spaced on the log scale of the lag range, respectively. Different maximum lags of up to 21 days were assessed to explore the lag patterns of temperature, but the initial analysis indicated relatively short lag patterns; therefore, the present study focused on maximum lags of 0, 2, 4, and 7 days. We compared changes in standardized NPX at low (5^th^ and 10^th^ percentile: −2.1 and 1.9 °C) and high (90^th^ and 95^th^ percentile: 27.9 and 29.5 °C) temperatures with reference to the median temperature (17.7 °C), respectively. Proteins that were consistently significant at both percentiles under low or high temperatures across the four lag scenarios were considered as differentially expressed proteins (DEPs) and were classified into four groups: (i) downregulated with cold, (ii) upregulated with cold, (iii) downregulated with heat, and (iv) upregulated with heat.

We also conducted various subgroup analyses in the whole case-cohort data set to explore effect modification by age, sex, self-rated health, education level, and heating use. To test the reliability of the results, we conducted sensitivity analyses by (i) changing knot placements (to 10^th^ and 90^th^ of the temperature distribution) in the exposure distributions, (ii) changing spline function specification (to integer function) in the lag dimensions in the DLNMs of temperature, and (iii) excluding samples showing potential QC warnings where incubation controls deviated by ≥0.3 from the median values for all samples on any plate and any proteins (see Table S1 for the distribution).

To assess the biological relevance of the temperature-related DEPs, we identified proteins that are also associated with baseline systolic blood pressure (SBP) and prospectively recorded incident IHD cases (ICD-10 codes: I20–I25), two major health outcomes that were known to be strongly related with temperature, using generalized linear regression (adjusted for age, age^2^, sex, study area, fasting time, fasting time^2^, plate ID, education, smoking, alcohol drinking, and physical activity) and Cox regression with the Prentice pseudopartial likelihood method^35^ (similar adjustment but stratified by sex and study area), respectively. We compared the distributions of the DEPs identified in the primary analyses with the background distribution (in proportion) of proteins included in the Olink panel according to established biological pathways, and conducted enrichment analyses involving the Kyoto Encyclopedia of Genes and Genomes (KEGG) and Reactome databases using DAVID (Database for Annotation, Visualization and Integrated Discovery) to assess their biological function.

All statistical analyses were conducted in the R software (version 4.3.0) by using the *mgcv* (version 1.8-42) and *dlnm* (version 2.4.7) packages. The Benjamini–Hochberg false discovery rate (FDR) and the more stringent Bonferroni-significance thresholds were used to control for multiple testing in the main and sensitivity analyses, respectively.

## Results

Of the 3926 participants included, the mean (standard deviation [SD]) age was 58.0 (19.0) years, 53.8% were female, 61.9% reported use of heating during winter, and day 0 mean temperature varied widely from −27.4 to 34.3 °C with an overall median (interquartile range [IQR]) of 17.7 (15.1) °C (Table 1; Figure S2). Higher temperature was associated with a higher percentage of females, higher household income, lower proportions of smokers and drinkers, lower levels of SBP and diastolic blood pressure (DBP), and higher RH. Similar patterns of association were observed among the 2006 subcohort participants (Table S2).

**Table 1:** Baseline Characteristics of 3926 Participants by Tertiles of Ambient Temperature on the Day of Blood Sample Collection^a^

**Characteristics**	**Tertiles of temperature**			**All** (*N* = 3926)
	T1 (*n* = 1293)	T2 (*n* = 1339)	T3 (*n* = 1294)	
age, years	58.0 (19.0)	58.0 (20.0)	57.0 (19.0)	58.0 (19.0)
female, %	52.0	52.2	57.2	53.8
urban, %	43.3	50.8	54.3	49.5
no formal or primary school, %	55.1	54.1	54.4	54.6
annual household income <10,000 yuan, %	34.4	33.5	28.3	32.1
household heating, %	77.2	64.5	44.1	61.9
current regular smoker, %	32.7	30.8	26.9	30.1
weekly regular drinker, %	16.6	15.4	13.4	15.2
BMI, kg/m^2^	23.8 (4.6)	23.9 (4.9)	23.5 (4.7)	23.7 (4.8)
waist circumference, cm	81.5 (14.1)	82.2 (14.0)	81.0 (15.0)	81.6 (14.5)
SBP, mmHg	140.5 (32.0)	135.0 (33.0)	129.0 (30.0)	134.5 (32.5)
DBP, mmHg	80.0 (16.5)	78.5 (14.5)	76.0 (14.5)	78.0 (15.0)
self-rated poor health, %	11.1	12.8	10.2	11.4
respiratory diseases, %	12.5	11.1	13.0	12.1
diabetes, %	11.1	11.9	10.7	11.3
cancer, %	0.5	0.4	1.0	0.6
fasting time, h	3.0 (2.0)	3.0 (2.0)	3.0 (2.0)	3.0 (2.0)
Time to blood process, h	10.8 (13.9)	10.4 (13.9)	9.2 (12.5)	9.9 (13.5)
time in storage, days	75.0 (67.0)	68.0 (73.0)	73.0 (75.0)	71.0 (71.0)
relative humidity, %	57.2 (28.4)	67.5 (24.3)	75.6 (17.5)	68.2 (26.2)
mean temperature, °C	5.0 (8.1)	17.2 (5.0)	26.0 (4.0)	17.3 (15.5)

a Continuous variables are presented in median (interquartile range), and categorical variables are presented as percentage. Abbreviations: BMI = body mass index, SBP = systolic blood pressure, and DBP = diastolic blood pressure.

Overall, 1364 (46.7%) proteins were significantly associated (at 5% FDR) with temperature at lag 0 (i.e., on the day of blood collection), which declined with longer cumulative lags to 1290 at lag 0–7, with a greater proportional reduction for proteins associated with cold than with heat (Table 2). Across all four lag models, 949 DEPs were consistently shown to be associated with temperature, with 216 downregulated and 171 upregulated with cold and 656 downregulated and 114 upregulated with heat. Among the subcohort participants, the patterns of association were similar (Table 2). Although there were fewer (*n* = 673) DEPs across the four lag models, they largely (95%) overlapped with those in the overall case-cohort data set with highly comparable effect sizes (Figure S3). Sensitivity analyses with knot placements at 10th and 90th percentile temperatures, integer function for the lag–response associations, and exclusions of samples showing potential QC warnings yielded highly consistent results, both overall and in the subcohort data set (Table S3).

**Table 2:** Summary of Statistically Significant Associations between Proteins and Ambient Temperature at Lag 0, Lag 0–2, Lag 0–4, and Lag 0–7 Days after Multiple Testing Adjustment^a^

**Model**	**Total**	**Association with cold**^b^		**Association with heat**^c^	
		Downregulated	Upregulated	Downregulated	Upregulated
**Whole case-cohort(*N* = 3926)**^d^					
Lag 0	1364 (46.7)	299 (10.2)	217 (7.4)	721 (24.7)	127 (4.3)
Lag 0–2	1309 (44.8)	240 (8.2)	204 (7.0)	728 (24.9)	137 (4.7)
Lag 0–4	1315 (45.0)	254 (8.7)	188 (6.4)	748 (25.6)	125 (4.3)
Lag 0–7	1290 (44.1)	235 (8.0)	187 (6.4)	736 (25.2)	132 (4.5)
Significant across models	949 (32.5)	216 (7.4)	171 (5.9)	656 (22.4)	114 (3.9)
**Subcohort(*N* = 2006)**^e^					
Lag 0	943 (32.3)	133 (4.6)	137 (4.7)	579 (19.8)	94 (3.2)
Lag 0–2	917 (31.4)	113 (3.9)	105 (3.6)	603 (20.6)	96 (3.3)
Lag 0–4	904 (30.9)	104 (3.6)	74 (2.5)	636 (21.8)	90 (3.1)
Lag 0–7	871 (29.8)	110 (3.8)	59 (2.0)	620 (21.2)	82 (2.8)
Significant across models	673 (23.0)	94 (3.2)	56 (1.9)	520 (17.8)	76 (2.6)

a *N* (%) is presented. Percentage is the proportion of significant hits out of the 2923 Olink proteins.

b Both changes in proteins at the 5^th^ and 10^th^ percentile vs median temperatures are statistically significant after multiple test adjustment.

c Both changes in proteins at the 90^th^ and 95^th^ percentile vs median temperatures are statistically significant after multiple test adjustment.

d Models are adjusted for relative humidity, region, year of sample collection, fasting time, fasting time^2^, age, age^2^, sex, hour of blood collection, hours to blood processing, and case ascertainment status. Day 0 temperatures (°C) at the 5^th^, 10^th^, 50^th^, 90^th^, and 95^th^ percentiles are −2.1, 1.9, 17.7, 27.9, and 29.5, respectively.

e Models are adjusted for relative humidity, region, year of sample collection, fasting time, fasting time^2^, age, age^2^, sex, hour of blood collection, and hours to blood processing. Day 0 temperatures (°C) at the 5^th^, 10^th^, 50^th^, 90^th^, and 95th percentiles are −2.2, 2.1, 17.6, 27.8, and 29.5, respectively.

**Figure 1:**
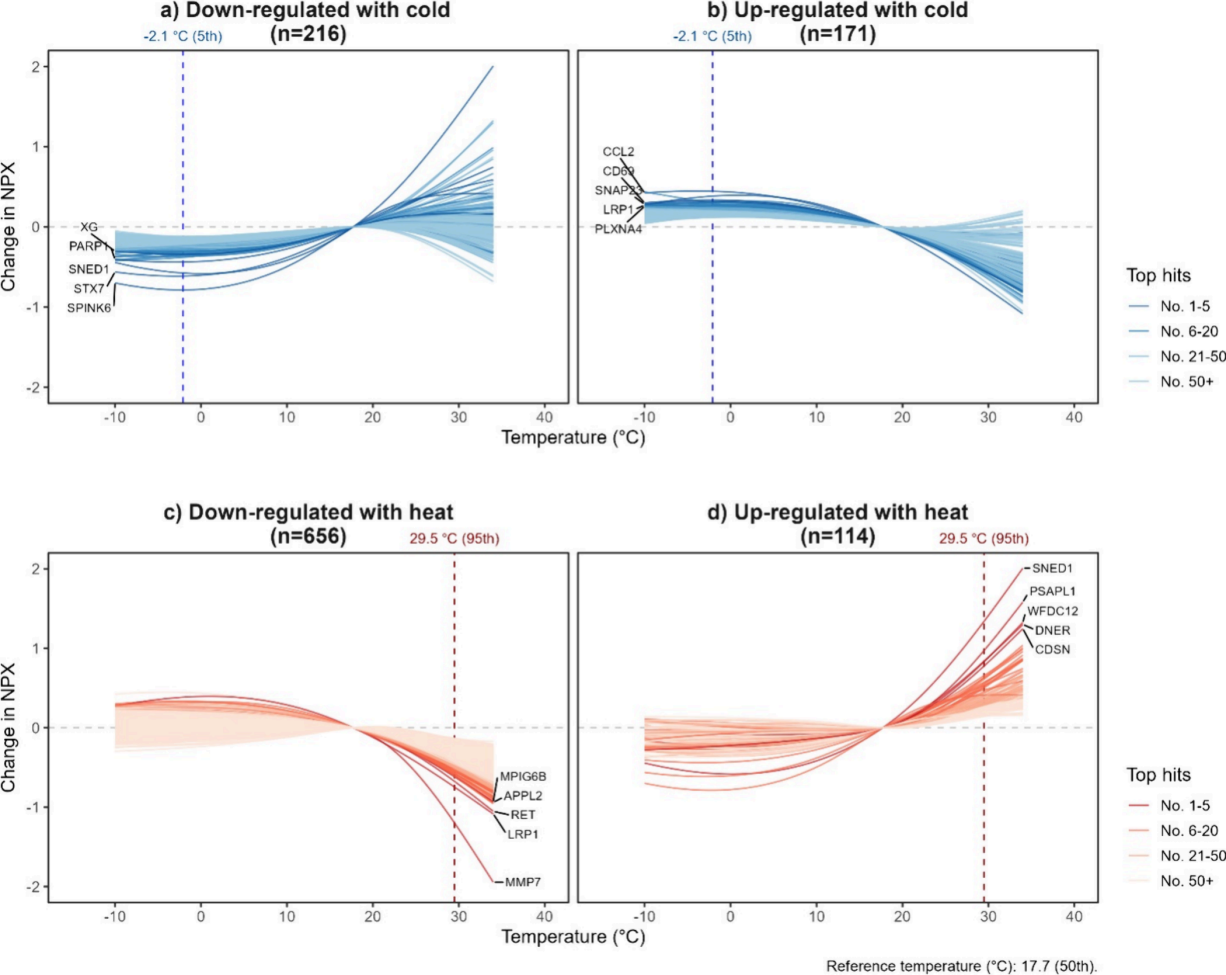
Cumulative exposure–response relationship over lag 0–2 days of DEPs found to be (a) downregulated with cold, (b) upregulated with cold, (c) downregulated with heat, and (d) upregulated with heat in the whole case-cohort. Abbreviations: DEP = differentially expressed protein and NPX = Normalized Protein eXpression.

Among the 949 DEPs identified, the majority demonstrated nonlinear relationships across the temperature ranges examined (Figure 1), with similar patterns among the subcohort participants (Figure S4). For DEPs that were down- or upregulated with cold, most showed significant departures from the null at around 10 °C and attenuated below 5 °C, with slightly stronger effect sizes for some of the downregulated (range: −0.116 to −0.787) than the upregulated proteins (range: 0.117 to 0.445) DEPs (Figure 1A and 1B; Table S4). In contrast, DEPs associated with heat, up- or downregulated, typically demonstrated stronger effect sizes than those associated with cold and broadly linear associations above the median temperature reference point (17.7 °C) (Figure 1C and 1D; Table S4). Generally, most DEPs showed lag patterns between lags 0 and 2 (Figure S5).

**Figure 2:**
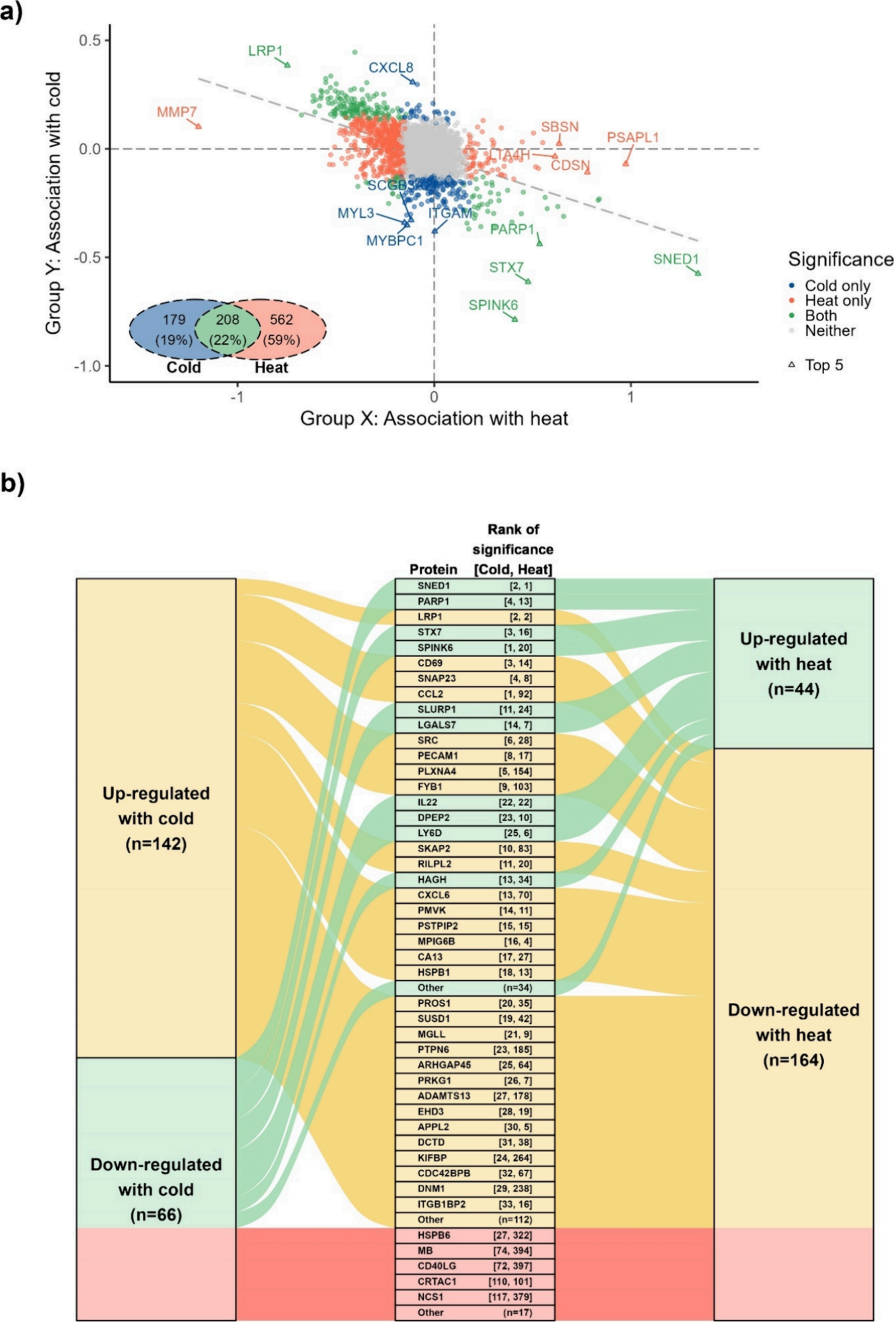
Comparison of (a) the associations of cold and heat with 949 DEPs and (b) 208 DEPs with coherent associations identified from the primary analysis. For associations with cold, changes in NPX at the 5th percentile vs median temperature are presented; for associations with heat, changes in NPX at the 95th percentile vs median temperature are presented. The top 20% of the coherent DEPs in each category are shown. Abbreviations: DEP = differentially expressed proteins and NPX = Normalized Protein eXpression.

Overall, there were 208 overlapping proteins between cold- and heat-related DEPs, which constituted 54 and 27% of all cold- and heat-related DEPs, respectively, with most overlapped DEPs showing unidirectional associations across the temperature range (i.e., DEPs upregulated with cold were downregulated with heat, and vice versa) (Figure 2). Among the most statistically significant overlapping DEPs, there are SNED1, PARP1, STX7, SPINK6, and SLURP1 that were downregulated with cold and upregulated with heat and LRP1, CD69, SNAP23, CCL2, and SRC that were upregulated with cold and downregulated with heat (Figure 2B). On the other hand, proteins such as HSPB6, MB, CD40LG, CRTAC1, and NCS1 showed inverted U-shaped associations with temperature (Figure 2B). Other nonoverlapping DEPs with strong statistical signals and potential biological relevance include ITGAM, MYBPC1, and MYL3 that were downregulated with cold; CXCL8, LACRT, and NADK that are upregulated with cold; MMP7, ASPN, and MMP1 that were downregulated with heat; and PSAPL1, CDSN, and LTA4H that were upregulated with heat.

**Figure 3:**
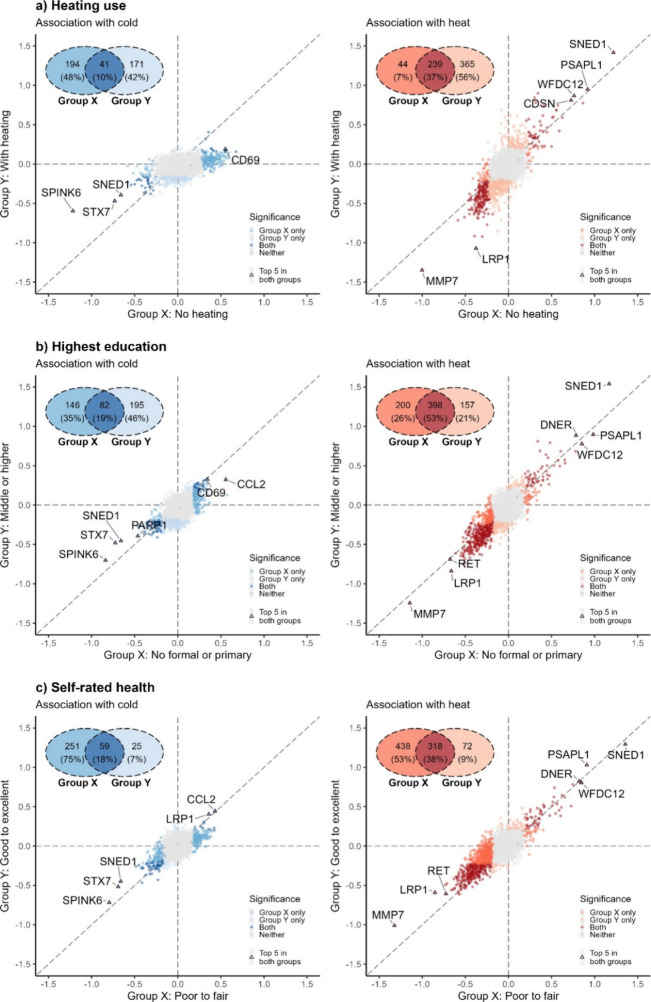
Subgroup analyses of the temperature–protein associations by (a) heating use, (b) highest education, and (c) self-rated health. For associations with cold, changes in NPX at the 5^th^ percentile vs median temperature are presented; for associations with heat, changes in NPX at the 95^th^ percentile vs median temperature are presented. Abbreviations: DEP = differentially expressed proteins and NPX = Normalized Protein eXpression.

In the subgroup analyses, the most striking effect modifications were by heating use, with signficiantly weaker associations with cold and considerably stronger associations with heat in participants who reported using heating at home (Figure 3). Importantly, the flattened associations with cold persisted in those with heating, but the associations were more likely to be linear among those without heating (Figure S6). Likewise, the protein associations were somewhat stronger with cold among participants with lower education, poor self-rated health, and lower body mass index (BMI) and household income and stronger with heat among those with poor self-rated health (Figure 3; Figure S7). There were little differences in these associations among age- or sex-specific subgroups (Figure S7). The temperature exposure patterns were broadly consistent across subgroups, except that participants with heating were more likely to be exposed to a colder temperature than those without heating (Table S5).

**Figure 4:**
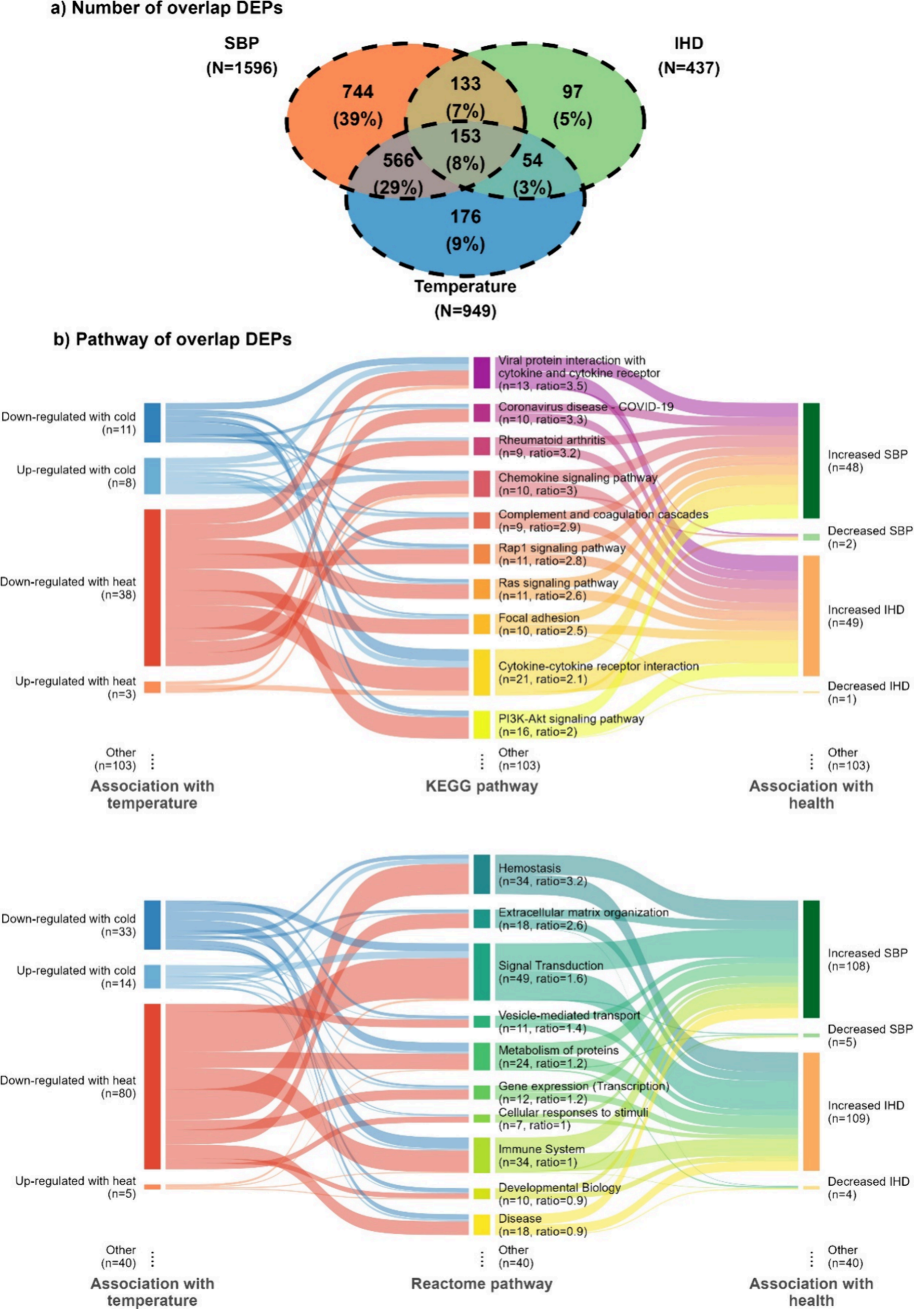
DEPs associated with temperature, SBP, and IHD and the top 10 KEGG and Reactome pathways. Pathways are ordered by the ratio of proportions of overlap DEPs of temperature, SBP, and IHD over that of background proteins. Abbreviations: DEP = differentially expressed protein, SBP = systolic blood pressure, IHD = ischemic heart disease, and KEGG = Kyoto Encyclopedia of Genes and Genomes.

Of the 949 DEPs identified, 773 (81%) were also significantly associated with SBP or IHD, with 153 overlapping proteins across all three sets (Figure 4A). Among the 773 overlapping DEPs, 577 were downregulated with heat and positively associated with SBP (*n* = 684) or IHD (*n* = 196) (Table S6). Among the 153 proteins with three-way overlaps, there were greater-than-expected proportions of FDR-significant hits in several KEGG pathways, including viral protein interaction with cytokine and cytokine receptor, COVID-19, chemokine signaling, coagulation cascades, and cytokine–cytokine receptor interaction (Figure 4B; Figure S8A), and in Reactome pathways, particularly hemostasis, extracellular matrix organization, and signal transduction (Figure 4B; Figure S8B). Enrichment analysis of the 153 three-way overlapping DEPs demonstrated evidence of enrichment of pathways related to platelet activation, signaling and aggregation, degranulation, hemostasis, and chemokines (Figure S9). Consistently, the 949 DEPs showed evidence for platelet activation for proteins upregulated with cold and downregulated with heat; signaling by interleukins for proteins upregulated with heat; signal transduction, platelet activation, signaling and aggregation, lipid and atherosclerosis, and immune system for proteins downregulated with heat (Figure S9).

## Discussion

To our knowledge, this is the first study to assess the acute impact of nonoptimal temperatures on large-scale plasma proteome in an East Asian population. Among the 2923 proteins examined, we found that 949 (32%) DEPs were associated with cold or heat across multiple cumulative-lag models of 0 to 7 days after extensive adjustment for potential confounders and multiple testing. Most of the DEPs showed nonlinear associations with ambient temperature, chiefly reflecting an attenuation below ∼5 °C and a broadly linear association with heat (above 17.7 °C). The strength of temperature–protein associations differed by heating use, self-rated health, BMI, and socioeconomic factors. Importantly, over 80% of the DEPs were also associated with SBP and IHD, which are known to be associated with nonoptimal temperatures.

### Comparison with Previous Studies

Several previous studies have reported short-term associations of ambient temperature with certain inflammatory protein markers, including several interleukins (e.g., IL6 and IL8) and high-sensitive CRP (hs-CRP),^36^ but few have included a wide spectrum of proteomic data as in the present study. A study of 1115 older people (mean age 70.4 years) in Germany reported short- to medium-term (lag 0–1 to lag 0–55) moving averages of temperature to be inversely and linearly associated with higher levels of 17 to 59 inflammatory proteins (out of 71 assayed using an early Olink platform).^26^ The associations were chiefly found with longer lag (lag 0–27 and lag 0–55) and in individuals with pre-existing cardiovascular diseases or those aged 70 years or older.^26^ A recent study of 2961 US adults assayed ∼6300 protein markers using an aptamer-based SomaScan platform and found 1904 proteins to be significantly associated with 5 year annual and seasonal average ambient temperature after FDR correction. However, the analyses were minimally adjusted for potential confounders (age, sex, and race) and the reported associations of 5 year temperature with SBP (inverse) and DBP (positive)^27^ and differed from the well-established inverse associations of ambient temperature with BP.

In the German and US studies, there were 17 and 236 proteins significantly associated with temperature, respectively, that were also associated with temperature in CKB. Of these, we found 2 (CXCL5 and CXCL6) and 135 proteins, respectively, in the German and US studies showing directionally consistent associations with temperature in CKB. The differences in study findings between these three studies may reflect differences in physiological and behavioral adaptation between populations, in addition to time frames of temperature exposures (short in CKB versus longer in other studies) and the range and number of proteins captured. While some plasma proteins could have relatively long half-lives (e.g., 19 days for albumin), many are short-lived (<1 day), are sensitive to acute bodily changes, and are constantly produced and metabolized.^37^ Therefore, we focused on the short-term (0–7 days) impact of temperature on the plasma proteome and found evidence of a relatively short lag structure for most proteins. In particular, there was a gradual reduction of the number of significant associations when extending the lag days, but most of the attenuated associations showed relatively weak statistical significance even in shorter-lag models, whereas the top significant hits (with the smallest *p*-values) remained robust across models. Although longer time-lagged associations are plausible, averages across long time frames may only crudely approximate the general neighborhood climate condition, which may not be appropriate for capturing the acute biological impact of variations in ambient temperature. Since the previous studies did not assess adaptation factors that could alter personal exposure to temperature (e.g., heating or air-conditioning use), it is difficult to assess the extent to which their findings were influenced by these factors.

In CKB, we found that a significant proportion of the temperature–protein associations, including the overlapping DEPs noted above (e.g., CXCL6), were nonlinear, in contrast to the linear associations reported in the German study.^26^ For CKB, a likely reason for the attenuation in the associations at low temperatures is the use of heating or other cold-related adaptation that prevents personal experienced temperature exposure to drop below a certain level.^38^ In contrast, while reliable domestic heating in Germany should be more widely available than in China, the predominant composition of elderly and individuals with pre-existing disease (who have poorer adaptability) in the German study may explain the linear inverse associations, although there are also other potential issues with overadjustment by having both SBP and DBP as covariates in the models.^26^

The findings in CKB suggest that the overall strength of associations of proteins with cold is attenuated by about 50% by home heating versus no heating, despite the greater absolute temperature differences when comparing the 5^th^ percentile (i.e., cold exposure) to median temperature in the subgroups (with heating: 20.3 °C vs without heating: 17.3 °C). Importantly, similar exposure–response patterns have been found between ambient temperature and BP in CKB, with a strong linear inverse association (−0.6 mmHg SBP per 1 °C higher ambient temperature above 10 °C) that leveled-off below 5–10 °C among participants with city-wide district heating.^39^ The generally colder climate around participants with heating versus without heating (median [IQR] = 15.5 [7.0–22.4] vs 20.2 [12.3–25.9]°C) may have also resulted in other behavioral or biological acclimatization to cold, which may also explain the apparently stronger effects of heat in the former, who may be less resilient to heat. Overall, the findings of this study highlight the importance of heating on attenuation of the effect of cold on plasma protein levels. However, the low use of air-conditioning in the present study population in 2004–2008 precluded any assessment of the impact of this on heat in CKB. Moreover, the lack of air-conditioning may explain the broadly linear associations between heat and plasma proteins.

### Disease-Relevant Biological Pathways and Proteins

While cardiovascular diseases are largely consistently associated with nonoptimal temperatures,^5^ the present study demonstrated that about 80% of the temperature-related DEPs were directionally consistently associated with SBP or IHD risk. For example, heat was associated with lower levels of MMP7, LRP1, RET, and MPIG6B, which were also associated with lower levels of SBP in CKB; cold was associated with higher levels of CCL2, LRP1, and CD69, which were associated with higher levels of SBP in CKB. For IHD, while we have previously shown higher levels of 13 proteins to be causally and positively associated with increased risk,^31^ four (CCL17, TFPI, F2R, ASGR1) of them were also found to be upregulated with cold, which are consistent with the widely reported winter surge in cardiovascular mortality and hospitalization related to low temperature.^40^

Both the overall list of 949 DEPs related to ambient temperature and the 153 overlaps with SBP and IHD have been implicated in multiple pathways linking temperature with the cardiovascular impact of temperature. The DEPs include well-established chemokines (e.g., CCL2, CXCL5, CXCL3, and PPBP), enzymes (e.g., MMP1 and MMP7), and interleukins (e.g., IL22 and IL15) involved in inflammation-, immunity-, and infection related pathways with etiological relevance to a wide range of diseases beyond cardiovascular disease.^41−43^ Key DEPs such as MPIG6B^44^ and MGLL^45^ were involved in hemostasis and platelet activation, signaling, aggregation, and degranulation, suggesting a potential role of temperatures in hemorrhage or thrombosis and ischemic vascular issues. For example, previous mechanistic studies suggest that cold exposure prolonged coagulation times *in vitro* and bleeding times *in vivo* in rabbits,^46^ while heat exposure induced hyperaggregability in platelet-rich plasma *in vitro*.^47^ Consistently, our findings show that MPIG6B, a novel inhibitory receptor on the surface of platelets against platelet aggregation and activation,^44^ was upregulated with cold and downregulated with heat. We also found that temperature influences blood lipids or lipid-related pathways (e.g., MMP1 and MMP7)^48^ and chemokines (e.g., CCL2 and CXCL3),^49^ both of which pathologically contribute to the development of plaques and atherosclerosis.^50^ Such findings are consistent with previous mechanistic evidence suggesting temperature acclimation of brown adipose tissue in humans^51^ and cold-induced changes in lipid and fatty acid composition observed in pig skeletal muscle.^52^ Additionally, a population-based research among 2.8 million US adults reported significant seasonal variation of the plasma lipid profile (e.g., with higher levels of low-density lipoprotein cholesterol in winter), implying the relevance of temperature in mediating lipid metabolism.^53^ Similarly, prior *in vivo* studies revealed that mRNA and protein levels of chemokines such as CCL2 and CCL5 were temperature-dependent in mice.^54,55^

In addition to the links with SPB and blood lipids, other temperature-associated proteins found in this study were linked to many other plausible mechanisms. For example, proteins that were downregulated with cold and upregulated with heat included SNED1, a novel extracellular matrix (ECM) protein found to be a promoter of breast cancer metastasis,^56^ while occupational heat exposure was linked to elevated female breast cancer risk in a Spain study;^57^ PARP1, which has an important role in DNA damage detection and repair,^58^ while hypothermia has been found to delay DNA damage repair in *in vitro* studies;^59^ SPINK6, a potent inhibitor of serine proteases that are essential for influenza A viruses infection in the airways,^60^ while cold temperatures are known to be associated with higher respiratory infection risk; SLURP1, which exerts anti-inflammatory effects and support the maintenance of the physiological and structural integrity of the skin,^61^ which may reflect a protective mechanism against heat. Among proteins that are upregulated with cold and downregulated with heat, LRP1 plays an important role in lipid homeostasis and acts as a master regulator of tau uptake and spread,^62^ which have significant implications on obesity and risk of CVD and dementia; CD69, CCL2, and MMP7 may play a role in immune responses involving memory T cells^63,64^ and alveolar epithelial injuries,^65^ which are consistent with the higher risks of infection with cold temperature.

### Strengths and Limitations

This is one of the largest investigations of the impact of nonoptimal temperatures on the human plasma proteome, quantified using a well-established Olink platform covering an extensive range of proteins of potential biological relevance. We integrated state-of-the-art molecular and environmental epidemiology approaches to examine the nonlinear exposure–lag associations using DLNM with distinct advantages over the use of moving or long-term averages employed in previous studies. We applied stringent criteria to focus on DEPs consistently associated with heat or cold across multiple lag models after extensive adjustment of key confounders, providing a selective list of temperature-related proteins. However, this study also had several limitations. First, as in most temperature epidemiology studies, we examined residence-based ambient temperature instead of directly measuring personal temperature exposure, which was infeasible on the scale of the original cohort. The exposure misclassification, likely nondifferential, could reduce the power to detect relatively weak associations. Nonetheless, given the large number of associations with DEPs at high levels of statistical significance, the findings cannot be attributed to chance. Second, we used a cross-sectional study design to link measured protein levels with ambient temperatures prior to and concurrent with the time of blood collection. Although the cross-comparison of multiple lag models enabled some assessment of temporality, future studies measuring plasma proteome across multiple time points are required to assess the causal relevance of these associations. Third, while the present study examined a wide spectrum of temperature exposure across China, extreme cold or heat tends to concentrate in certain regions, so there were less data and thus greater uncertainty at the two extreme ends of the exposure–response relationships. Therefore, the present report focused primarily on the top significant associations with relatively clear exposure–response relationships even at moderate cold or heat (i.e., 10^th^ to 90^th^ percentiles). Fourth, the present study focused on the mean daily temperature, whereas other temperature-related metrics, such as temperature variability and nighttime heat, should be investigated in future studies. Fifth, the large number of DEPs identified prevented us from discussing individual proteins in detail. However, the findings of this study provide an atlas of likely temperature-related proteins that can inform future studies. Fundamentally, this is an epidemiological study designed to explore the impact of temperature on a wide range of proteins and pathways and to generate hypotheses and signpost researchers for future studies, including mechanistic studies to understand the largely understudied mechanisms linking temperature to individual plasma proteins.

In Chinese adults, nonoptimal temperatures were associated with significantly higher and lower plasma levels of 949 proteins consistently across multiple lag models. While most of these proteins are associated with higher SBP and IHD, two conditions that have been strongly associated with temperature in many previous studies, we also identified several likely relevant pathways, including inflammation, platelet activation, and endothelial dysfunction. These shed light on the biological mechanisms of the health impact of temperature and inform identification of possible therapeutic targets that can be explored further in the prevention and treatment of CVD. Importantly, our study provided for the first time a novel atlas of temperature-related proteomic signatures in Chinese adults, which could inform not only further downstream experimental research but also future proteomics and epidemiological research on analytical strategies (e.g., adjustment for temperature) and clinical practices on standardizing biomarker measurement protocols (e.g., under controlled temperature conditions to ensure accuracy and reliability).

## Data Availability

Data from baseline, first and second resurveys, and disease follow-up are available under the CKB Open Access Data Policy to bona fide researchers. Sharing of genotyping data is constrained by the Administrative Regulations on Human Genetic Resources of the People’s Republic of China. Access to these and certain other data is available through collaboration with CKB researchers. Details of the CKB Data Sharing Policy are available at www.ckbiobank.org.

## Supporting Information Available

Figures S1–S9 and Tables S1–S6 with additional details on the map of the study areas in CKB; temperature distribution by study area; comparison of the proteome-wide associations with temperature in the whole case-cohort and subcohort samples; cumulative exposure–response relationship in the subcohort; clusters of lag–response relationship in the whole case-cohort; cumulative exposure–response relationship in participants with and without heating in the whole case-cohort; subgroup analyses of the temperature–protein associations; top 10 KEGG and Reactome pathways implicated in the DEPs associated with temperature, SBP, and/or IHD; downstream enrichment analyses; distribution of proteins with QC warnings; baseline characteristics of 2006 subcohort; sensitivity analyses of the temperature–protein associations; distribution of changes in DEPs; summary statistics of ambient temperature and DEPs in different subgroups; and comparisons of DEPs associated with temperature, SBP, and/or IHD (PDF)

## Notes

This research was funded in whole, or in part, by the Wellcome Trust [212946/Z/18/Z, 202922/Z/16/Z, 104085/Z/14/Z, and 088158/Z/09/Z]. For the purpose of Open Access, the author has applied a CC-BY public copyright license to any Author Accepted Manuscript version arising from this submission.

## Abbreviations

ASGR1: asialoglycoprotein receptor 1
ASPN: asporin
BMI: body mass index
BP: blood pressure
CCL17: C–C motif chemokine 17
CCL2: C–C motif chemokine 2
CD40LG: CD40 ligand
CD69: early activation antigen CD69
CDSN: corneodesmosin
CKB: China Kadoorie Biobank
CRTAC1: cartilage acidic protein 1
CRP: C-reactive protein
CVD: cardiovascular disease
CXCL3: C-X-C motif chemokine 3
CXCL5: C-X-C motif chemokine 5
CXCL6: C-X-C motif chemokine 6
CXCL8: C-X-C Motif chemokine 8
DAVID: Database for Annotation, Visualization and Integrated Discovery
DBP: diastolic blood pressure
DEP: differentially expressed protein
DLNM: distributed lag nonlinear model
DNA: DNA
ECMWF: European Centre for Medium Range Weather Forecasts
ERA5: fifth-generation European Centre for Medium Range Weather Forecasts (ECMWF) reanalysis database for global climate and weather
F2R: proteinase-activated receptor 1
FDR: false discovery rate
GAM: generalized additive model
hs-CRP: high-sensitivity C-reactive protein
HSPB6: heat shock protein beta-6
IHD: ischemic heart disease
IL15: interleukin-15
IL22: interleukin-22
IL6: interleukin-6
IL8: interleukin-8
IQR: interquartile range
ITGAM: integrin alpha-M
KEGG: Kyoto Encyclopedia of Genes and Genomes
LACRT: extracellular glycoprotein lacritin
LRP1: prolow-density lipoprotein receptor-related protein 1
LTA4H: leukotriene A-4 hydrolase
MB: myoglobin
MGLL: monoglyceride lipase
MMP1: interstitial collagenase
MMP7: matrilysin
MPIG6B: megakaryocyte and platelet inhibitory receptor G6b
MYBPC1: myosin-binding protein C, slow-type
MYL3: myosin light chain 3
NADK: NAD kinase
NCS1: neuronal calcium sensor 1
NPX: Normalized Protein eXpression
PARP1: poly[ADP-ribose] polymerase 1
PDGFA: platelet-derived growth factor subunit A
PDGFB: platelet-derived growth factor subunit B
PPBP: platelet basic protein
PSAPL1: proactivator polypeptide-like 1
QC: quality control
RET: proto-oncogene tyrosine-protein kinase receptor Ret
RH: relative humidity
SBP: systolic blood pressure
SD: standard deviation
SLURP1: secreted Ly-6/uPAR-related protein 1
SNAP23: synaptosomal-associated protein 23
SNED1: sushi, nidogen and EGF-like domain-containing protein 1
SPINK6: serine protease inhibitor Kazal-type 6
SRC: proto-oncogene tyrosine-protein kinase Src
STX7: syntaxin-7
TFPI: tissue factor pathway inhibitor
TNC: tenascin
