# Supplementary material for: Acute Impact of Nonoptimal Ambient Temperatures on Plasma Levels of 3000 Proteins in Chinese Adults
Source: Environ Sci Technol. 2025 Mar 4;59(10):4868–82. doi: 10.1021/acs.est.4c13020 (PMC11924237; doi:10.1021/acs.est.4c13020)
Supplement: Supplementary file 1 — es4c13020_si_001.pdf [file es4c13020_si_001.pdf]

Supporting Information

Acute impact of non-optimal ambient temperatures on plasma levels of 3000 proteins in Chinese adults

Yi Tong Guo[1,2], Mohsen Mazidi[2], Neil Wright[2], Pang Yao[2], Baihan Wang[2], Yue Niu[3], Xi Xia[4,5,6], Xia Meng[3], Cong Liu[3], Robert Clarke[2], Kin Bong Hubert Lam[2], Christiana Kartsonaki[2], Iona Millwood[2], Yiping Chen[2], Ling Yang[2], Huaidong Du[2], Canqing Yu[7,8,9], Dianjianyi Sun[7,8,9], Jun Lv[7,8,9], Liming Li[7,8,9], Junshi Chen[10], Maxim Barnard[2], Xiaocao Tian[11], Kin Fai Ho[1*], Ka Hung Chan[2*], Antonio Gasparrini[12†], Haidong Kan[3,13†], Zhengming Chen[2†] on behalf of the China Kadoorie Biobank Study group[#]

1 JC School of Public Health and Primary Care, The Chinese University of Hong Kong, Hong Kong SAR, China

2 Clinical Trial Service Unit and Epidemiological Studies Unit, Nuffield Department of Population Health, University of Oxford, Oxford, OX3 7LF, UK

3 School of Public Health, Key Lab of Public Health Safety of the Ministry of Education and NHC Key Lab of Health Technology Assessment, Fudan University, Shanghai, 200433, China

4 Department of Occupational and Environmental Health, School of Public Health, Xi'an Jiaotong University Health Science Center, Xi'an, 710061, China

5 Key Laboratory of Environment and Genes Related to Diseases, Ministry of Education, Xi'an, 710000, China

6 School of Public Health, Shaanxi University of Chinese Medicine, Xi'an, 030001, China

7 Department of Epidemiology and Biostatistics, School of Public Health, Peking University Health Science Center, Beijing, 100871, China

8 Peking University Center for Public Health and Epidemic Preparedness & Response, Beijing, 100871, China

9 Key Laboratory of Epidemiology of Major Diseases (Peking University), Ministry of Education, Beijing, 100071, China

10 China National Center for Food Safety Risk Assessment, Beijing, 100000, China

11 Qingdao Center of Disease and Control and Prevention, Qingdao, 266000, China

12 Environment & Health Modelling (EHM) Lab, Department of Public Health Environments and Society, London School of Hygiene & Tropical Medicine, London, WC1 E7H, UK

13 Children's Hospital of Fudan university, National Center for Children's Health, Shanghai, 200433, China

*Joint corresponding authors; †Joint senior authors; #Members of the CKB Collaborative Group are shown in the Appendix

Address for correspondence

Kin-Fai Ho: JC School of Public Health and Primary Care, The Chinese University of Hong Kong, Shatin, New Territories, HKSAR, China. E-mail address: **kfho@cuhk.edu.hk**;

Ka Hung Chan: Clinical Trial Service Unit and Epidemiological Studies Unit, Nuffield Department of Population Health, University of Oxford, Oxford, UK. E-mail address: **kahung.chan@ndph.ox.ac.uk**.

This file includes:

A total of 42 pages.
Figure S1 to S9.
Table S1 to S6.

Figure S1. Study sites of the China Kadoorie Biobank

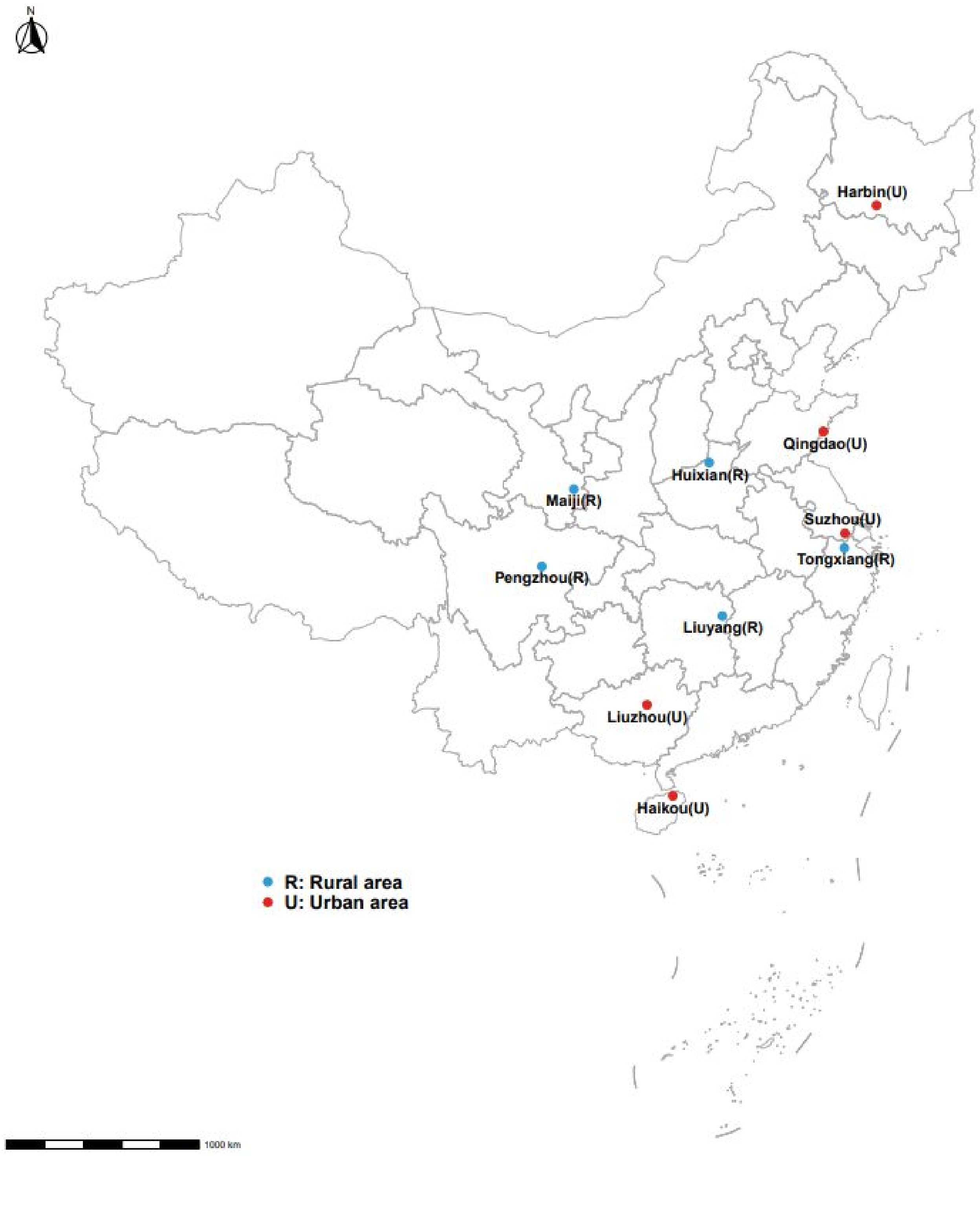

Figure S2. Temperature distribution by study area

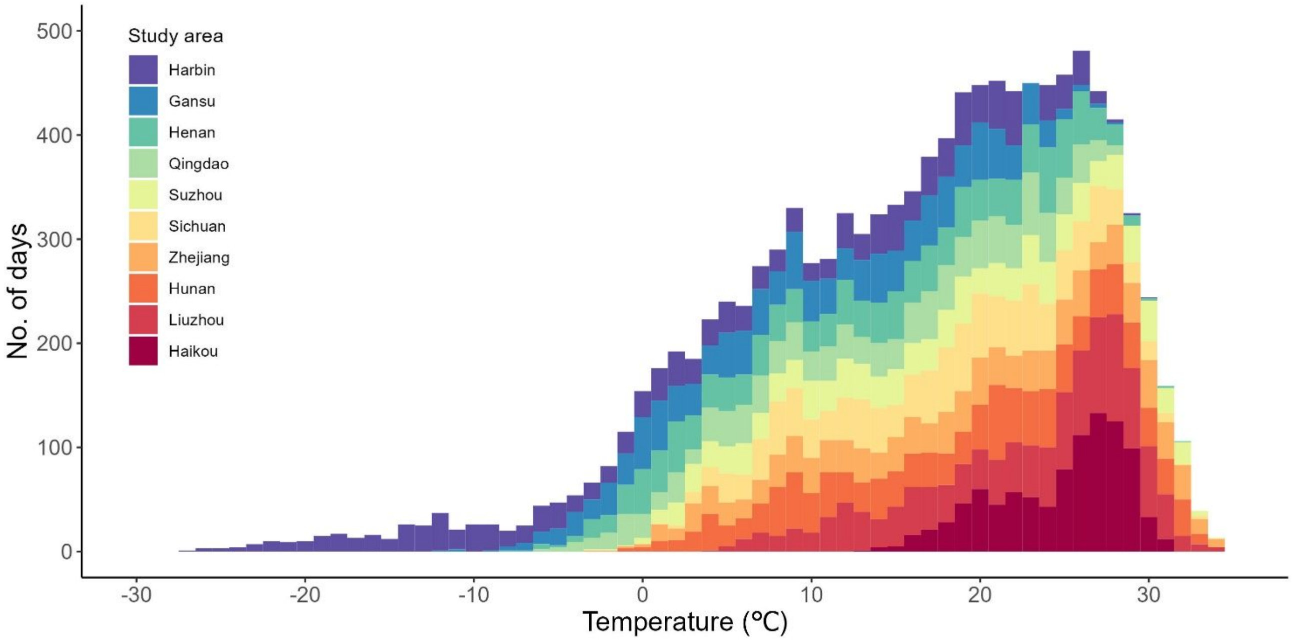

Figure S3. Comparison of the proteome-wide associations with temperature in the whole case-cohort and sub-cohort samples.

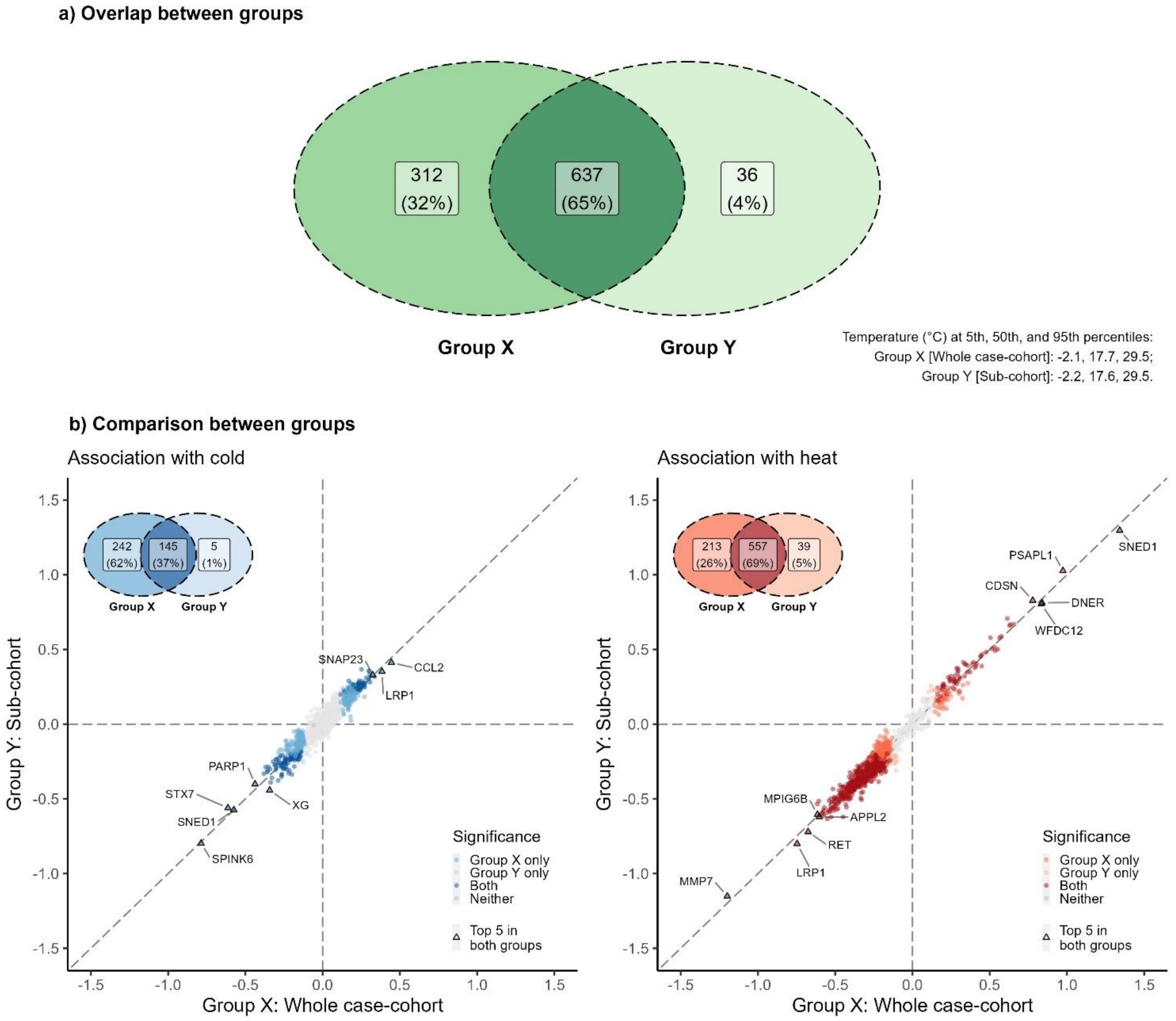

For associations with cold, changes in NPX at 5th percentile vs. median temperature are presented; for associations with heat, changes in NPX at 95th percentile vs. median temperature are presented.

Figure S4. Cumulative exposure-response relationship over lag 0-2 days of DEPs found to be a) down-regulated with cold, b) up-regulated with cold, c) down-regulated with heat, and d) up-regulated with heat in the sub-cohort.

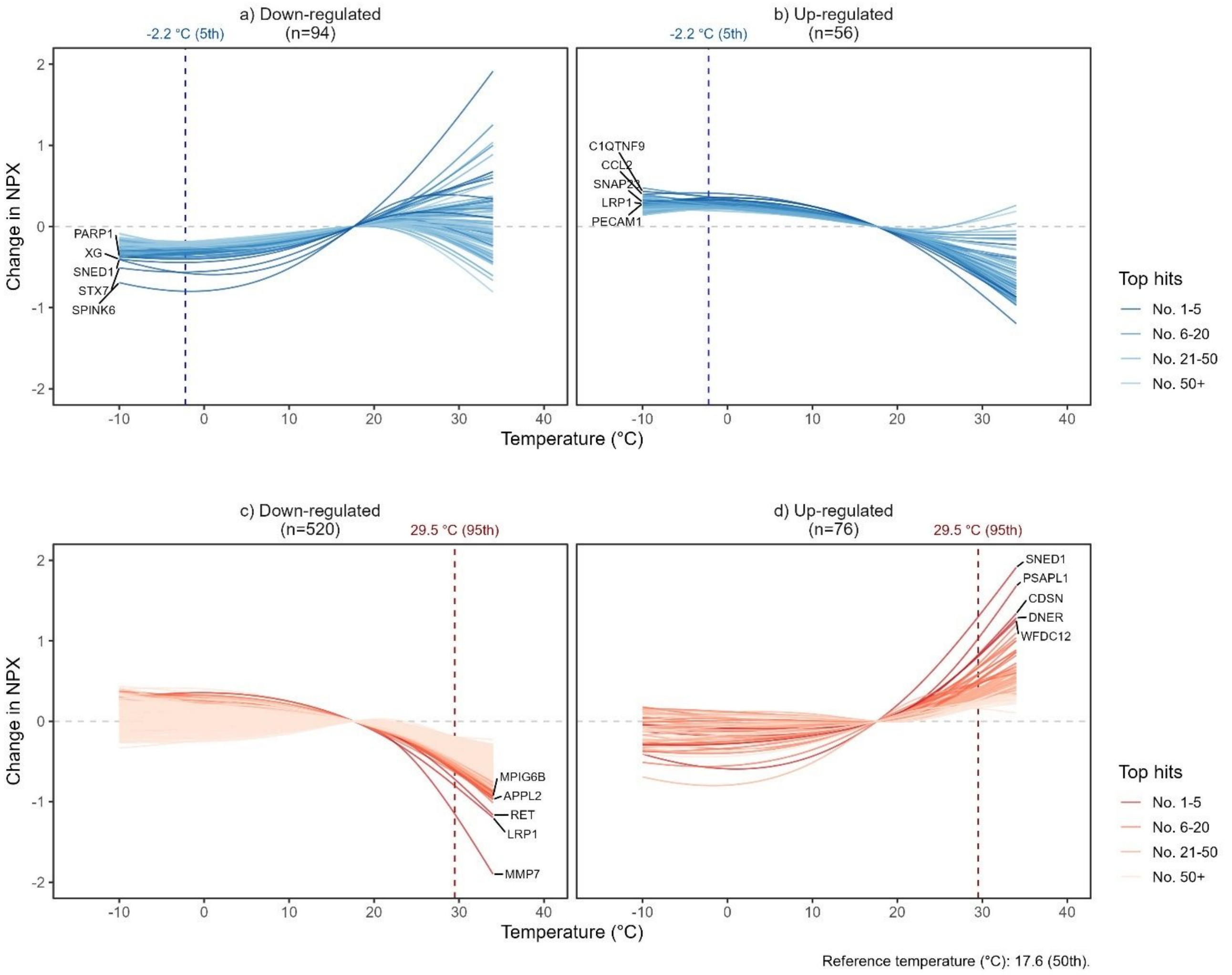

Abbreviation: DEP, differentially expressedX, Normalized Protein eXpression.

Figure S5. Clusters of lag-response relationship of DEPs found to be a) down-regulated with cold, b) up-regulated with cold, c) down-regulated with heat, and d) up-regulated with heat in the whole case-cohort.

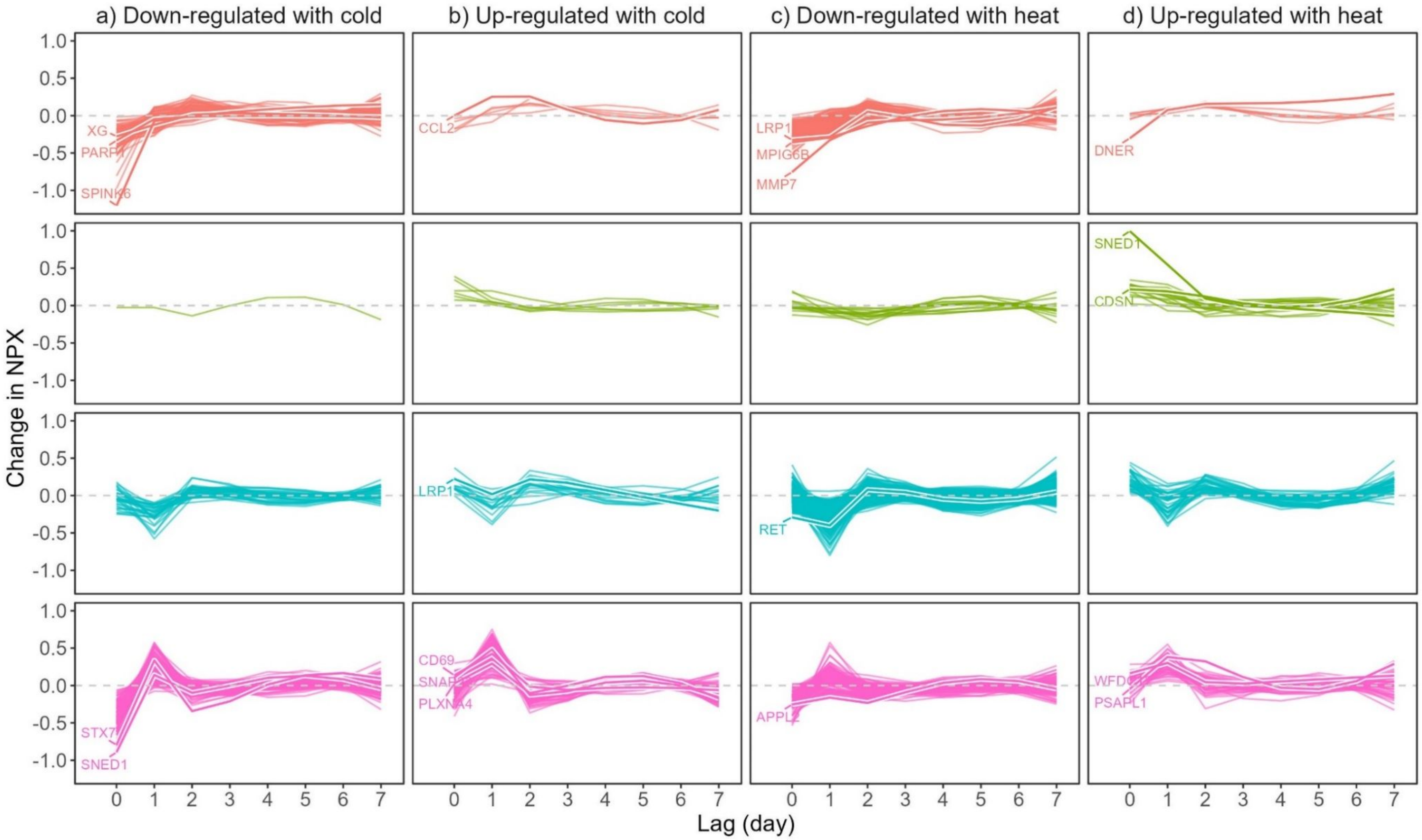

Abbreviation: DEP, differentially expressed protein; NPX, Normalized Protein eXpression.

Figure S6. Cumulative exposure-response relationship over lag 0-2 days of DEPs in participants with and without heating in the whole case-cohort.

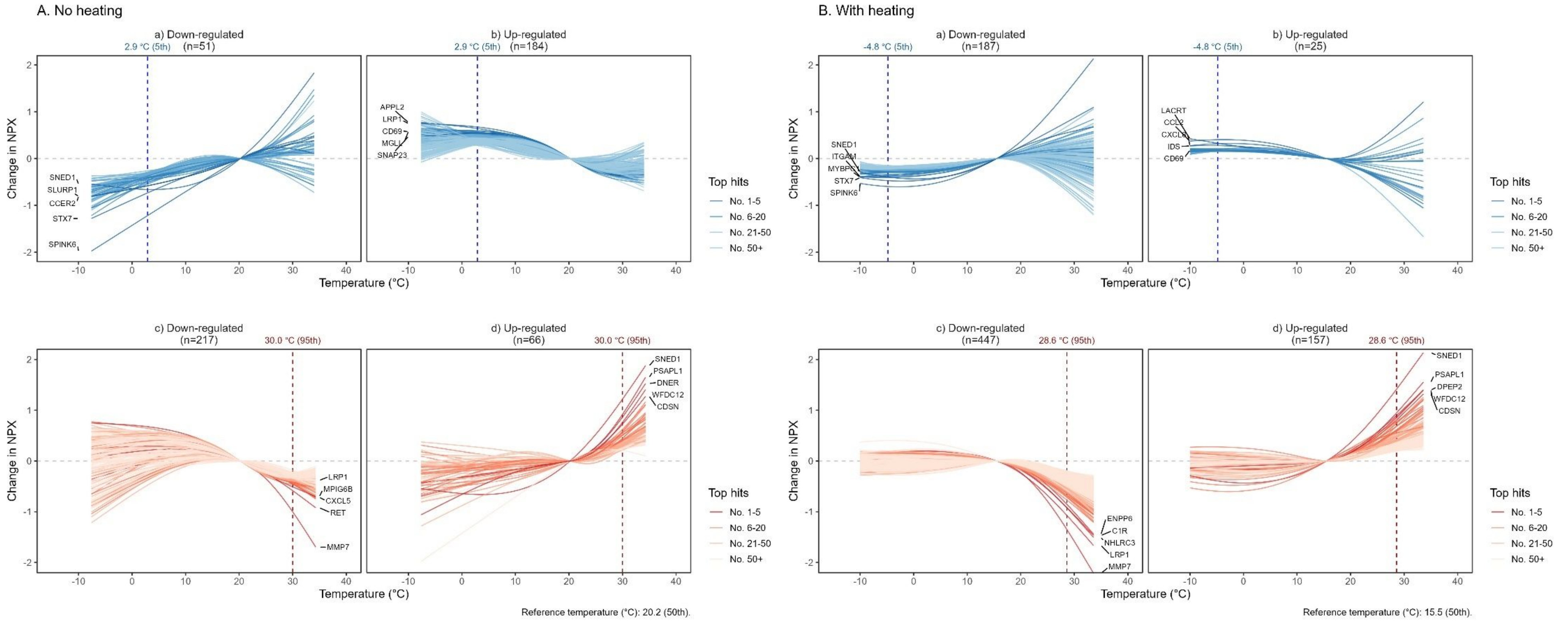

Abbreviation: DEP, differentially expressed protein; NPX, Normalized Protein eXpression.

Figure S7. Subgroup analyses of the temperature-protein associations by a) age, b) sex, c) annual household income, and d) BMI.

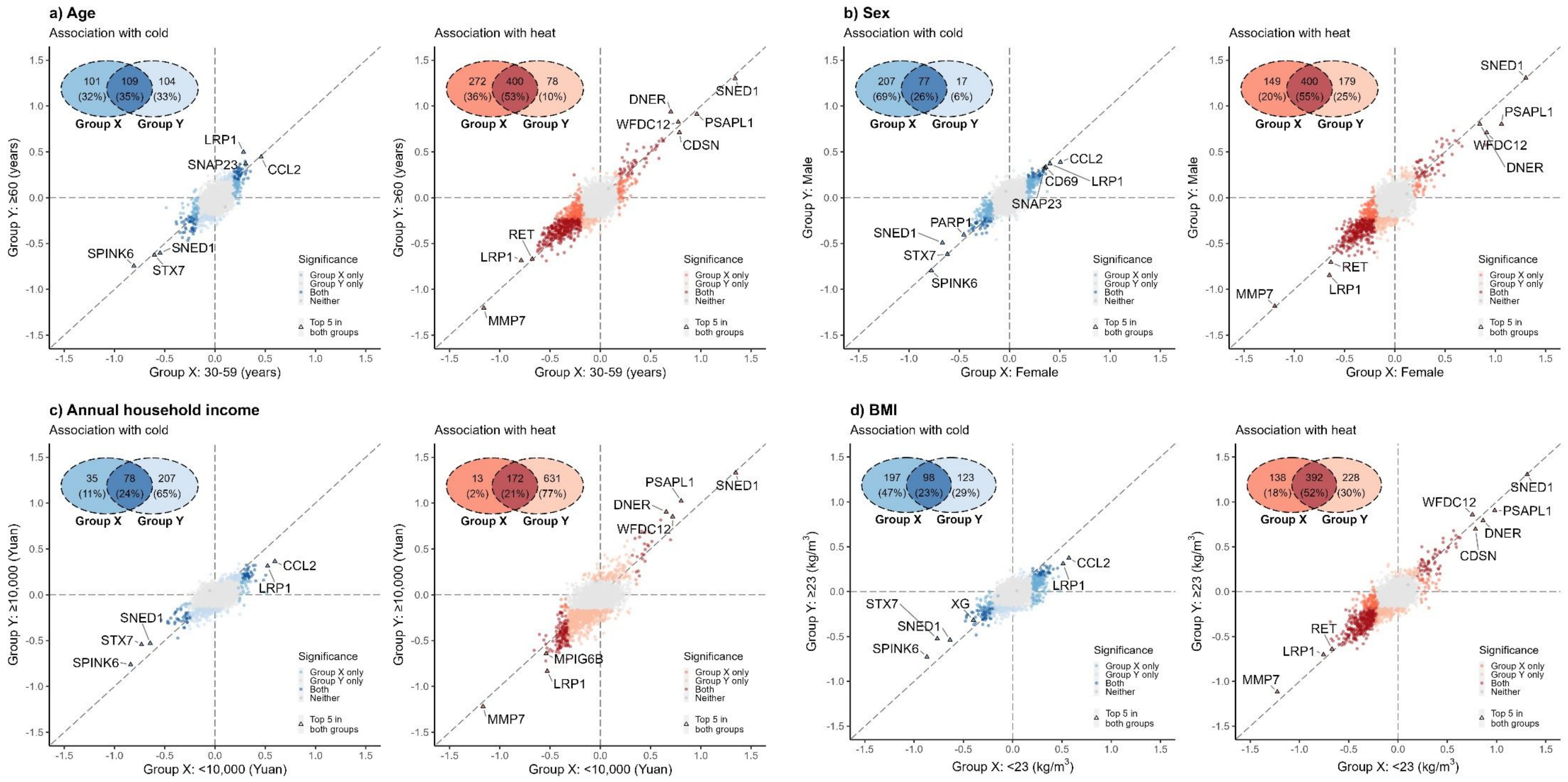

For associations with cold, changes in NPX at 5th percentile vs. median temperature are presented; for associations with heat, changes in NPX at 95th percentile vs. median temperature are presented. Abbreviation: DEP=differentially expressed proteins; NPX=Normalized Protein eXpression.

Figure S8. Top-10 KEGG and Reactome pathways implicated in the DEPs associated with temperature, SBP, and/or IHD.

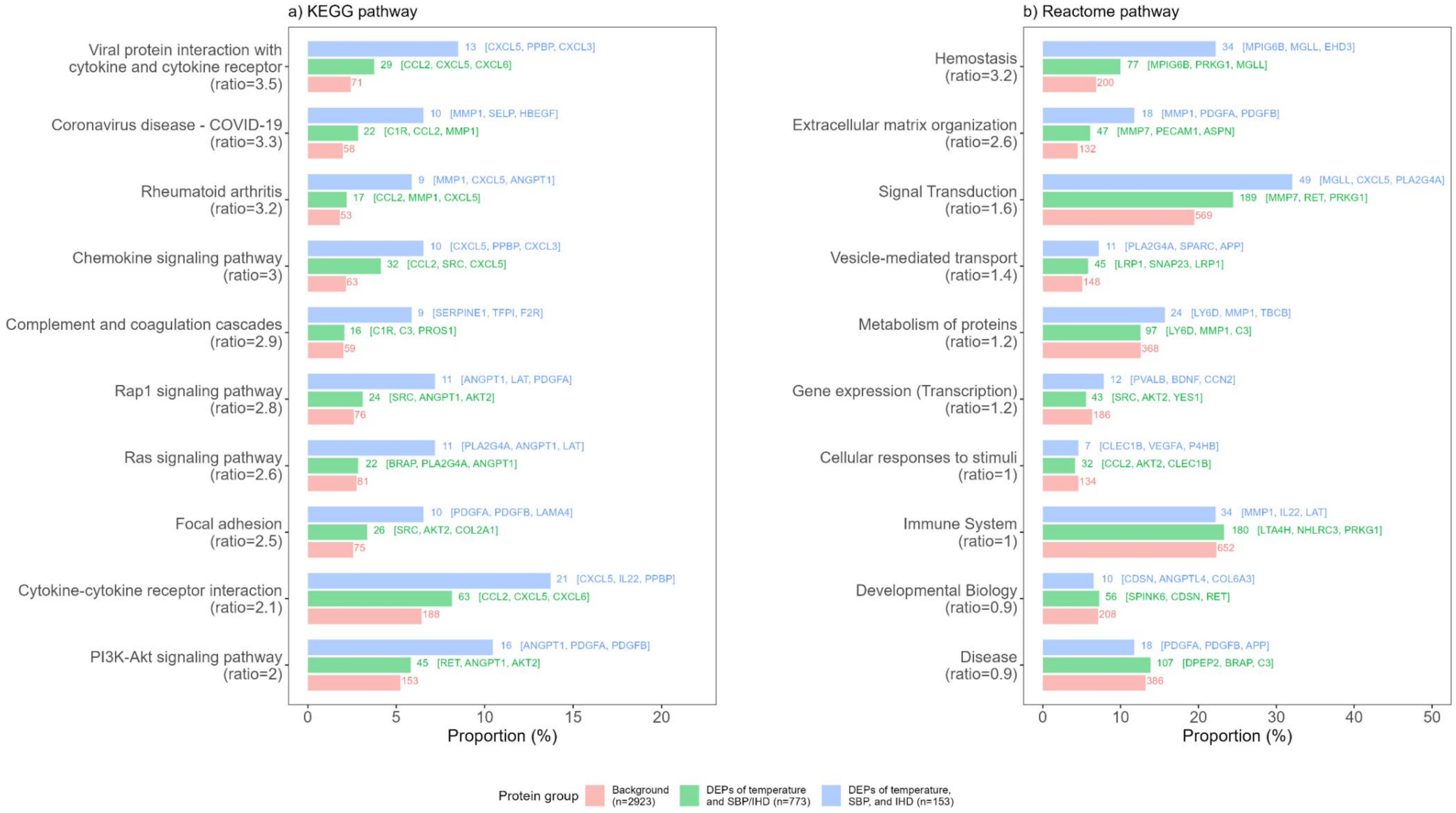

Pathways are ordered by the ratio of proportions of overlap DEPs of temperature, SBP, and IHD over that of background proteins. Abbreviation: DEP, differentially expressed protein; SBP, systolic blood pressure; IHD, ischaemic heart disease; KEGG, Kyoto Encyclopedia of Genes and Genomes.

Figure S9. Downstream enrichment analyses on a) 153 overlapping DEPs associated with temperature, SBP, and IHD and b) 949 DEPs associated with temperature in the whole case-cohort participants.

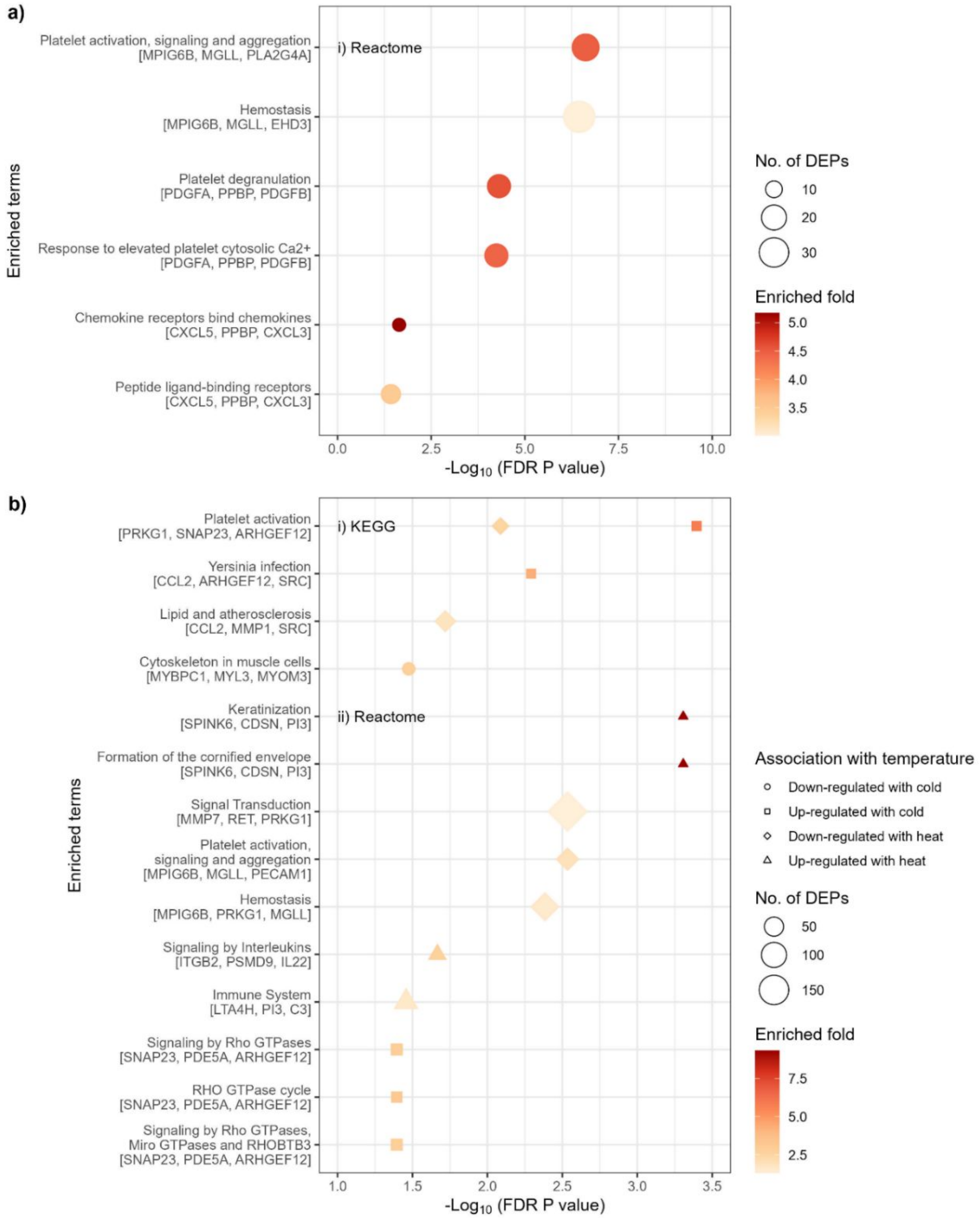

Abbreviation: DEP, differentially expressed protein; SBP, systolic blood pressure; IHD, ischaemic heart disease; FDR, false discovery rate; KEGG, Kyoto Encyclopedia of Genes and Genomes.

Table S1. Distribution of proteins with QC warnings among 3926 participants

% of values with QC warnings	n (%) of proteins
<1%	1 186 (50.9)
<2%	598 (25.7)
<3%	452 (19.4)
<4%	687 (29.5)

Abbreviation: QC=quality control.

Table S2. Baseline characteristics of 2006 sub-cohort participants by tertile of ambient temperature on the day of blood sample collection

Characteristics	Tertiles of temperature			All (N=2 006)
	T1 (n=665)	T2 (n=678)	T3 (n=663)	
Age (year) [a]	50.0 (16.0)	51.0 (16.8)	50.0 (16.0)	50.0 (16.0)
Female, %	59.8	62.2	64.4)	62.2
Urban, %	47.7	52.7	53.1	51.1
No formal or primary school, %	48.0	46.6	48.0	47.5
Annual household income <10,000 Yuan, %	27.1	29.1	21.7	26.0
Household heating, %	69.5	55.5	37.6	54.2
Current regular smoker, %	29.3	23.0	23.5	25.3
Weekly regular drinker, %	17.7	15.2	13.0	15.3
BMI (kg/m^2) [a]	23.6 (4.3)	24.0 (5.0)	23.2 (4.4)	23.6 (4.5)
Waist circumference (cm) [a]	79.8 (13.2)	80.7 (13.5)	78.5 (13.5)	79.8 (13.2)
SBP (mmHg) [a]	132.0 (24.5)	127.8 (28.5)	122.0 (25.0)	127.5 (26.5)
DBP (mmHg) [a]	78.5 (14.0)	77.0 (14.0)	75.5 (14.0)	77.0 (14.0)
Self-rated poor health, %	8.1	8.6	8.3	8.3
Respiratory diseases, %	10.4	9.4	10.7	10.2
Diabetes, %	7.1	7.2	5.1	6.5
Cancer, %	0.3	0.3	1.4	0.6
Fasting time (hour) [a]	3.0 (4.0)	3.0 (4.0)	3.0 (2.5)	3.0 (3.0)
Time to blood process (hour) [a]	9.6 (14.1)	10.4 (13.7)	8.4 (12.6)	9.4 (13.4)
Time in storage (day) [a]	79.0 (66.0)	64.0 (63.0)	68.0 (80.0)	69.0 (71.0)
Relative humidity (%) [a]	59.4 (28.0)	66.2 (24.9)	74.1 (17.8)	67.8 (25.8)
Mean temperature (°C) [a]	5.5 (7.6)	17.6 (5.1)	26.2 (3.8)	17.6 (15.6)

Abbreviation: BMI, body mass index; SBP, systolic blood pressure; DBP, diastolic blood pressure.

[a] Median (interquartile range).

Table S3. Sensitivity analyses of the temperature-protein associations at different lags among 3,926 participants in whole study.

Model	Total	Association with cold [a]		Association with heat [b]	
		Down-regulated	Up-regulated	Down-regulated	Up-regulated
Two knots placed at the 10th and 90th percentile temperatures for the exposure-response dimension					
Lag 0	1 373 (47.0)	296 (10.1)	176 (6.0)	758 (25.9)	143 (4.9)
Lag 0-2	1 295 (44.3)	234 (8.0)	152 (5.2)	759 (26.0)	150 (5.1)
Lag 0-4	1 289 (44.1)	240 (8.2)	132 (4.5)	780 (26.7)	137 (4.7)
Lag 0-7	1 237 (42.3)	216 (7.4)	117 (4.0)	764 (26.1)	140 (4.8)
Significant across models	972 (33.3)	199 (6.8)	109 (3.7)	696 (23.8)	124 (4.2)
Using integer function for the lag dimension					
Lag 0	1 364 (46.7)	299 (10.2)	217 (7.4)	721 (24.7)	127 (4.3)
Lag 0-2	1 309 (44.8)	240 (8.2)	204 (7.0)	728 (24.9)	137 (4.7)
Lag 0-4	1 315 (45.0)	254 (8.7)	188 (6.4)	748 (25.6)	125 (4.3)
Lag 0-7	1 278 (43.7)	229 (7.8)	190 (6.5)	730 (25.0)	129 (4.4)
Significant across models	947 (32.4)	214 (7.3)	173 (5.9)	654 (22.4)	114 (3.9)
Removing values with QC warnings					
Lag 0	1 362 (46.6)	296 (10.1)	220 (7.5)	710 (24.3)	136 (4.7)
Lag 0-2	1 311 (44.9)	248 (8.5)	198 (6.8)	718 (24.6)	147 (5.0)
Lag 0-4	1 311 (44.9)	250 (8.6)	185 (6.3)	746 (25.5)	130 (4.4)
Lag 0-7	1 258 (43.0)	231 (7.9)	184 (6.3)	705 (24.1)	138 (4.7)
Significant across models	943 (32.3)	213 (7.3)	169 (5.8)	649 (22.2)	119 (4.1)
Bonferroni-PC adjustment					
Lag 0	631 (21.6)	112 (3.8)	78 (2.7)	376 (12.9)	65 (2.2)
Lag 0-2	605 (20.7)	94 (3.2)	70 (2.4)	378 (12.9)	63 (2.2)
Lag 0-4	614 (21.0)	104 (3.6)	61 (2.1)	388 (13.3)	61 (2.1)
Lag 0-7	563 (19.3)	90 (3.1)	56 (1.9)	357 (12.2)	60 (2.1)
Significant across models	459 (15.7)	89 (3.0)	53 (1.8)	332 (11.4)	54 (1.8)

Abbreviation: QC, quality control; PC, principal component.

Count (percentage) is presented. Percentage is the proportion of significant hits out of the 2,923 Olink proteins.

Models are adjusted for relative humidity, region, year of sample collection, fasting time, fasting time^2, age, age^2, sex, hour of blood collection, hours to blood processing, and case ascertainment status.

[a] Changes in proteins at 5th percentile (-2.1 °C) vs. median (17.7 °C) temperature.

[b] Changes in proteins at 95th percentile (29.5 °C) vs. median (17.7 °C) temperature.

Table S4. Distribution of changes in DEPs associated with temperature in the whole case-cohort

Group	Mean	SD	Min	25th	50th	75th	Max
Down-regulated with cold [a]	-0.203	0.081	-0.787	-0.233	-0.177	-0.153	-0.116
Up-regulated with cold [a]	0.194	0.050	0.117	0.162	0.180	0.212	0.445
Down-regulated with heat [b]	-0.287	0.113	-1.199	-0.355	-0.255	-0.201	-0.132
Up-regulated with heat [b]	0.315	0.189	0.143	0.195	0.248	0.357	1.341

Abbreviation: DEP=differentially expressed protein; SD=standard deviation; NPX=Normalized Protein eXpression.

Models are adjusted for relative humidity, region, year of sample collection, fasting time, fasting time^2, age, age^2, sex, hour of blood collection, hours to blood processing, and case ascertainment status.

[a] Changes in NPX at 5th percentile (-2.1 °C) vs. median (17.7 °C) temperature.

[b] Changes in NPX at 95th percentile (29.5 °C) vs. median (17.3 °C) temperature.

Table S5. Summary statistics of ambient temperature on the day of blood sample collection and proteins significantly associated with temperature in different subgroups of whole case-cohort participants.

Subgroup	n (%) of participants [a]	Temperature percentiles (°C)			n (%) of significant proteins [b]		
		5th	50th	95th	Total	Not overlap	Overlap
Sex							
Female	2 112 (53.8)	-2.0	17.9	29.5	673 (23.0)	227 (7.8)	446 (15.3)
Male	1 814 (46.2)	-2.4	17.7	29.3	621 (21.2)	175 (6.0)	446 (15.3)
Age (years)							
30-59	2 128 (54.2)	-2.3	17.4	29.3	761 (26.0)	294 (10.1)	467 (16.0)
≥60	1 798 (45.8)	-1.6	18.2	29.4	603 (20.6)	136 (4.7)	467 (16.0)
BMI (kg/m^3)							
<23	1 593 (40.6)	-0.8	18.1	29.5	646 (22.1)	183 (6.3)	463 (15.8)
≥23	2 333 (59.4)	-2.5	17.4	29.1	748 (25.6)	285 (9.8)	463 (15.8)
Self-rated health							
Poor to fair	2 235 (56.9)	-2.3	17.9	29.5	880 (30.1)	513 (17.6)	367 (12.6)
Good to excellent	1 691 (43.1)	-2.5	17.3	29.3	441 (15.1)	74 (2.5)	367 (12.6)
Highest education							
No formal or primary	2 142 (54.6)	-0.5	17.9	29.3	696 (23.8)	235 (8.0)	461 (15.8)
Middle or higher	1 784 (45.4)	-3.2	17.4	29.4	745 (25.5)	284 (9.7)	461 (15.8)
Annual household income (Yuan)							
<10,000	1 260 (32.1)	-1.3	17.8	29.2	250 (8.6)	23 (0.8)	227 (7.8)
≥10,000	2 666 (67.9)	-1.8	17.8	29.6	933 (31.9)	706 (24.2)	227 (7.8)
Heating use							
No heating	1 494 (38.1)	2.9	20.2	30.0	391 (13.4)	92 (3.1)	299 (10.2)
With heating	2 432 (61.9)	-4.8	15.5	28.6	743 (25.4)	444 (15.2)	299 (10.2)

Abbreviation: BMI, body mass index.

[a] %, percentage out of the 3 926 participants.

[b] %, percentage out of the 2 923 OLINK proteins.

Table S6. Comparisons of DEPs associated with temperature, SBP, and/ or IHD (n=773)

Uniprot ID	Symbol	Association with cold (n=356) [a]		Association with heat (n=648) [a]		Association with SBP (n=719) [b]		Association with IHD (n=207) [c]	
		Change in NPX [d]	FDR P value	Change in NPX [e]	FDR P value	Change in SBP [f]	FDR P value	HR [f]	FDR P value
P09237	MMP7	0.102	1.00E-01	-1.199 *	4.70E-97	3.715	8.80E-22	-	-
P06280	GLA	0.009	9.40E-01	-0.506 *	3.30E-16	3.758	2.90E-23	-	-
Q12805	EFEMP1	0.085	1.70E-01	-0.419 *	3.90E-14	3.289	1.50E-13	-	-
Q07108	CD69	0.335 *	8.90E-13	-0.526 *	2.50E-21	4.05	2.40E-23	-	-
P53814	SMTN	0.064	3.90E-01	-0.459 *	2.60E-14	2.873	7.30E-13	-	-
Q4KMG0	CDON	0.076	3.10E-01	-0.448 *	8.30E-13	3.677	9.70E-23	-	-
O00161	SNAP23	0.324 *	1.10E-11	-0.552 *	8.80E-23	3.911	2.60E-22	-	-
P13500	CCL2	0.445 *	1.30E-19	-0.404 *	2.00E-11	2.756	2.10E-12	-	-
P09104	ENO2	0.014	9.00E-01	-0.405 *	1.50E-10	2.855	4.30E-14	-	-
Q8WVQ1	CANT1	0.076	3.10E-01	-0.395 *	3.50E-10	3.425	1.30E-19	-	-
P11274	BCR	0.114	8.50E-02	-0.412 *	1.20E-10	2.397	2.60E-10	-	-
Q14BN4	SLMAP	-0.035	6.90E-01	-0.419 *	4.30E-12	2.486	1.10E-09	-	-
O60884	DNAJA2	0.118	7.10E-02	-0.383 *	1.80E-09	3.711	2.90E-23	-	-
P42785	PRCP	0.086	2.40E-01	-0.377 *	4.00E-09	4.172	2.70E-29	-	-
P12931	SRC	0.299 *	7.10E-09	-0.498 *	1.80E-16	3.042	3.40E-15	-	-
P16284	PECAM1	0.306 *	8.00E-09	-0.551 *	2.90E-19	2.886	1.30E-14	-	-
P52888	THOP1	-0.091	1.90E-01	-0.367 *	5.90E-09	3.375	1.50E-18	-	-
Q9HCM2	PLXNA4	0.305 *	1.20E-09	-0.343 *	8.10E-09	4.286	1.00E-27	-	-
O15117	FYB1	0.273 *	1.70E-08	-0.368 *	7.70E-11	3.643	2.10E-19	-	-
P25815	S100P	-0.048	5.70E-01	0.359 *	1.30E-08	2.693	1.00E-11	-	-
P19883	FST	-0.062	4.20E-01	-0.352 *	1.00E-08	2.358	3.10E-09	-	-
Q9H0P0	NT5C3A	0.112	9.50E-02	-0.423 *	3.30E-11	2.081	3.30E-08	-	-
P19021	PAM	-0.073	3.40E-01	-0.353 *	3.40E-08	2.924	2.60E-14	-	-
O75563	SKAP2	0.277 *	6.20E-08	-0.406 *	9.30E-12	3.338	2.10E-17	-	-
P23284	PPIB	0.079	2.80E-01	-0.349 *	4.80E-08	2.363	5.10E-10	-	-

Q969X0	RILPL2	0.282 *	7.70E-08	-0.528 *	2.90E-18	2.766	4.40E-13	-	-
P15692	VEGFA	-0.118	5.90E-02	-0.349 *	1.30E-08	2.15	1.10E-07	1.4	1.10E-08
Q96KN2	CNDP1	-0.062	4.20E-01	-0.332 *	1.00E-07	2.307	5.40E-09	-	-
P80162	CXCL6	0.270 *	2.20E-07	-0.424 *	2.00E-12	2.8	4.20E-13	-	-
Q15126	PMVK	0.269 *	2.30E-07	-0.576 *	2.80E-22	2.404	1.50E-09	-	-
Q9H939	PSTPIP2	0.263 *	2.40E-07	-0.545 *	9.20E-21	2.4	1.70E-09	-	-
O95825	CRYZL1	0.072	3.60E-01	-0.481 *	3.20E-14	1.985	1.60E-07	-	-
Q8TER0	SNED1	-0.575 *	1.80E-43	1.341 *	1.50E-147	-1.971	2.70E-07	-	-
Q16698	DECR1	0.093	1.90E-01	-0.336 *	1.90E-07	2.244	3.80E-09	-	-
P55210	CASP7	0.108	1.20E-01	-0.363 *	2.30E-08	1.945	1.90E-07	-	-
P09543	CNP	0.121	5.70E-02	-0.330 *	2.10E-07	2.183	1.10E-08	-	-
P78560	CRADD	0.03	7.60E-01	-0.333 *	2.00E-07	2.168	2.00E-08	-	-
P51692	STAT5B	0.102	1.40E-01	-0.336 *	2.90E-07	2.732	3.30E-13	-	-
Q16621	NFE2	0.118	7.60E-02	-0.356 *	4.60E-08	1.958	2.50E-07	-	-
P04792	HSPB1	0.263 *	4.90E-07	-0.570 *	1.20E-21	3.054	2.10E-15	-	-
Q53GL0	PLEKHO1	0.106	1.20E-01	-0.330 *	3.40E-07	2.577	7.20E-12	-	-
Q8TCD5	NT5C	0.041	6.50E-01	-0.446 *	2.10E-12	1.916	3.90E-07	-	-
Q07954	LRP1	0.384 *	1.50E-17	-0.747 *	7.20E-44	2.069	5.90E-07	-	-
Q9Y646	CPQ	-0.016	8.80E-01	-0.373 *	9.00E-09	1.923	4.10E-07	-	-
Q9BW04	SARG	0.108	1.10E-01	-0.368 *	9.00E-09	1.922	4.60E-07	-	-
Q9NYJ8	TAB2	0.097	1.50E-01	-0.397 *	2.60E-10	1.955	4.80E-07	-	-
P07225	PROS1	0.267 *	7.70E-07	-0.503 *	4.30E-16	3.557	1.10E-21	-	-
Q6UWL2	SUSD1	0.258 *	7.70E-07	-0.470 *	5.10E-15	2.987	2.60E-14	-	-
P13861	PRKAR2A	0.044	6.20E-01	-0.320 *	6.00E-07	2.859	4.30E-14	-	-
O75167	PHACTR2	0.115	7.90E-02	-0.330 *	3.40E-07	1.941	3.20E-07	-	-
Q9UBX1	CTSF	0.034	7.30E-01	-0.319 *	6.70E-07	2.995	1.70E-15	-	-
O94830	DDHD2	0.065	4.20E-01	-0.393 *	1.50E-09	1.854	7.40E-07	-	-
Q9BWV1	BOC	0.087	2.20E-01	-0.476 *	1.20E-14	1.958	7.40E-07	-	-
O94992	HEXIM1	0.072	3.60E-01	-0.324 *	7.50E-07	2.861	1.60E-14	-	-
Q9H6S3	EPS8L2	-0.280 *	2.40E-07	0.103	1.90E-01	1.923	7.00E-07	-	-

O60476	MAN1A2	-0.051	5.50E-01	-0.316 *	1.00E-06	2.307	1.60E-09	-	-
Q96K21	ZFYVE19	0.093	1.90E-01	-0.318 *	1.10E-06	2.335	4.80E-10	-	-
Q15814	TBCC	0.108	1.10E-01	-0.313 *	1.70E-06	2.731	4.40E-13	-	-
O60825	PFKFB2	0.118	6.90E-02	-0.307 *	2.00E-06	3.716	2.40E-23	-	-
P50053	KHK	0.004	9.80E-01	-0.308 *	2.10E-06	2.856	4.40E-14	-	-
Q12884	FAP	-0.124	5.50E-02	-0.307 *	2.30E-06	2.824	5.20E-14	-	-
Q9H910	JPT2	0.095	1.90E-01	-0.323 *	7.30E-07	1.796	1.90E-06	-	-
P29350	PTPN6	0.246 *	4.50E-06	-0.339 *	4.60E-08	3.28	1.50E-17	-	-
A6NI73	LILRA5	-0.105	1.30E-01	-0.305 *	3.20E-06	3.408	1.00E-19	-	-
P33316	DUT	0.073	3.40E-01	-0.326 *	4.20E-07	1.783	2.80E-06	-	-
Q9H4P4	RNF41	0.051	5.60E-01	-0.307 *	3.40E-06	3.432	6.80E-20	-	-
Q9UBQ7	GRHPR	0.014	9.00E-01	-0.302 *	3.40E-06	2.479	4.40E-11	-	-
Q99717	SMAD5	0.014	9.00E-01	-0.298 *	3.60E-06	3.826	4.00E-23	-	-
P14384	CPM	-0.024	8.20E-01	-0.299 *	3.90E-06	4.426	8.20E-32	-	-
Q96PL1	SCGB3A2	-0.328 *	1.20E-09	-0.118	1.30E-01	-1.748	4.30E-06	-	-
Q92619	ARHGAP45	0.246 *	7.70E-06	-0.452 *	6.20E-13	2.395	2.40E-10	-	-
Q13976	PRKG1	0.232 *	8.60E-06	-0.577 *	8.50E-23	2.183	3.60E-08	-	-
P19838	NFKB1	0.063	4.30E-01	-0.293 *	6.50E-06	2.827	3.00E-14	-	-
P49757	NUMB	0.122	5.70E-02	-0.294 *	6.70E-06	3.296	4.30E-18	-	-
P98161	PKD1	-0.087	1.90E-01	-0.280 *	5.20E-06	1.988	1.70E-06	-	-
Q8NDA2	HMCN2	0.085	2.40E-01	-0.294 *	7.20E-06	2.099	2.70E-08	-	-
O75506	HSBP1	0.102	1.30E-01	-0.324 *	4.50E-07	1.727	7.50E-06	-	-
Q8NEU8	APPL2	0.226 *	1.20E-05	-0.602 *	4.60E-25	2.039	4.00E-07	-	-
P32321	DCTD	0.237 *	1.20E-05	-0.490 *	1.50E-15	1.915	6.70E-07	-	-
Q9Y5S2	CDC42BPB	0.238 *	1.30E-05	-0.445 *	8.60E-13	1.95	3.50E-07	-	-
Q8WV92	MITD1	0.229 *	1.60E-05	-0.475 *	4.90E-15	2.897	1.40E-13	-	-
Q96EK5	KIFBP	0.253 *	5.50E-06	-0.294 *	6.80E-06	1.747	5.60E-06	-	-
O43399	TPD52L2	0.231 *	1.90E-05	-0.420 *	7.70E-12	2.898	4.30E-14	-	-
O94903	PLPBP	0.034	7.20E-01	-0.284 *	1.30E-05	2.429	1.60E-10	-	-
P01024	C3	0.073	3.10E-01	0.505 *	2.70E-17	1.706	1.50E-05	-	-

P04746	AMY2A	0.043	6.30E-01	-0.334 *	2.10E-07	-1.662	1.50E-05	-	-
Q13158	FADD	0.079	2.90E-01	-0.285 *	1.50E-05	1.95	3.40E-07	-	-
P15311	EZR	-0.064	4.00E-01	0.273 *	1.60E-05	2.215	1.10E-08	-	-
O94979	SEC31A	0.07	3.70E-01	-0.286 *	1.70E-05	2.623	1.60E-12	-	-
P20718	GZMH	-0.241 *	1.60E-05	-0.033	7.50E-01	1.85	2.00E-06	-	-
P42574	CASP3	0.232 *	2.70E-05	-0.480 *	1.80E-14	2.758	1.90E-13	-	-
Q96DE0	NUDT16	0.079	2.90E-01	-0.285 *	1.40E-05	1.763	4.20E-06	-	-
O75190	DNAJB6	0.116	7.60E-02	-0.278 *	2.00E-05	2.101	5.20E-08	-	-
Q6ZMJ2	SCARA5	-0.081	2.50E-01	-0.267 *	2.30E-05	3.755	1.60E-20	-	-
Q9UN19	DAPP1	0.230 *	3.20E-05	-0.406 *	8.70E-11	1.819	2.40E-06	-	-
Q9HD42	CHMP1A	0.069	3.80E-01	-0.278 *	2.20E-05	1.833	1.70E-06	-	-
O75791	GRAP2	0.223 *	3.60E-05	-0.454 *	7.20E-14	2.551	5.40E-11	-	-
O43639	NCK2	0.228 *	3.70E-05	-0.364 *	1.00E-08	1.925	5.30E-07	-	-
P06727	APOA4	-0.021	8.50E-01	-0.270 *	2.60E-05	2.141	3.90E-08	-	-
Q96B36	AKT1S1	0.039	6.70E-01	-0.276 *	2.80E-05	2.043	1.40E-07	-	-
P29279	CCN2	-0.078	2.60E-01	-0.349 *	5.80E-09	1.855	6.40E-06	1.3	3.60E-05
P19961	AMY2B	0.029	7.80E-01	-0.330 *	3.00E-07	-1.606	3.10E-05	-	-
P40818	USP8	-0.227 *	2.90E-05	-0.095	2.20E-01	1.881	2.50E-06	-	-
P14209	CD99	-0.213 *	9.10E-06	-0.086	2.10E-01	1.985	3.00E-05	1.4	1.30E-05
Q86X76	NIT1	-0.011	9.30E-01	-0.270 *	3.80E-05	2.328	1.20E-09	-	-
O14867	BACH1	0.063	4.40E-01	-0.277 *	3.90E-05	2.743	1.60E-13	-	-
O96007	MOCS2	-0.016	8.80E-01	-0.275 *	3.40E-05	1.744	6.20E-06	-	-
Q9HAV5	EDA2R	-0.183 *	1.20E-05	-0.031	6.70E-01	-	-	1.6	3.00E-05
Q96FZ7	CHMP6	0.052	5.60E-01	-0.274 *	4.30E-05	3.616	5.10E-23	-	-
P51617	IRAK1	0.046	6.20E-01	-0.275 *	4.80E-05	2.062	5.50E-08	-	-
Q8IXJ6	SIRT2	0.016	8.80E-01	-0.269 *	4.90E-05	2.801	1.40E-13	-	-
P62736	ACTA2	-0.101	8.70E-02	-0.232 *	7.40E-05	5.881	1.20E-37	1.5	6.00E-07
Q53H47	SETMAR	-0.111	1.00E-01	0.270 *	5.00E-05	3.208	1.20E-17	-	-
Q8NG06	TRIM58	0.063	4.40E-01	-0.271 *	5.00E-05	2.116	1.30E-08	-	-
Q92609	TBC1D5	0.103	1.30E-01	-0.267 *	5.00E-05	1.933	4.30E-07	-	-

O00194	RAB27B	0.208 *	7.70E-05	-0.461 *	7.40E-15	2.115	1.40E-07	-	-
P35443	THBS4	-0.056	5.00E-01	-0.264 *	5.20E-05	2.546	3.20E-11	-	-
Q9HB40	SCPEP1	-0.011	9.30E-01	-0.439 *	4.10E-12	1.556	5.30E-05	-	-
Q9UII2	ATP5IF1	0.079	2.90E-01	-0.267 *	6.20E-05	2.355	7.00E-10	1.3	2.00E-05
Q8NDI1	EHBP1	0.113	9.30E-02	-0.316 *	1.50E-06	1.536	5.40E-05	-	-
P52564	MAP2K6	0.102	1.40E-01	-0.398 *	5.40E-10	1.553	5.60E-05	-	-
Q96NZ9	PRAP1	-0.056	4.80E-01	-0.256 *	5.80E-05	4.295	7.90E-28	-	-
A0FGR8	ESYT2	0.03	7.70E-01	-0.289 *	1.00E-05	1.555	5.50E-05	-	-
P00736	C1R	0.219 *	1.10E-04	-0.616 *	9.50E-23	3.086	5.40E-17	-	-
Q14258	TRIM25	0.217 *	1.10E-04	-0.504 *	9.70E-16	2.173	7.70E-09	-	-
Q8N0X7	SPART	0.087	2.30E-01	-0.263 *	7.30E-05	2.181	1.70E-08	-	-
Q05315	CLC	-0.022	8.20E-01	0.263 *	2.10E-05	1.673	5.30E-05	-	-
O95721	SNAP29	0.211 *	1.20E-04	-0.409 *	2.60E-11	3.702	7.60E-22	-	-
Q9Y5X1	SNX9	0.024	8.20E-01	-0.261 *	8.80E-05	2.385	3.50E-10	-	-
Q6UWN8	SPINK6	-0.787 *	9.00E-70	0.410 *	5.20E-13	-1.538	1.30E-04	-	-
Q2M296	MTHFSD	0.066	4.10E-01	-0.294 *	9.60E-06	1.491	7.90E-05	-	-
Q99584	S100A13	-0.038	6.80E-01	-0.295 *	4.30E-06	1.56	8.60E-05	-	-
O15357	INPPL1	0.078	3.10E-01	-0.264 *	9.20E-05	1.797	2.60E-06	-	-
Q969Z4	RELT	-0.198 *	1.40E-04	-0.014	8.90E-01	2.473	2.00E-08	1.4	8.10E-06
P22004	BMP6	-0.107	1.20E-01	-0.264 *	8.70E-05	1.682	1.20E-05	-	-
Q9NZN5	ARHGEF12	0.211 *	1.50E-04	-0.537 *	4.90E-18	2.428	1.80E-10	-	-
Q9P1Z2	CALCOCO1	0.209 *	1.50E-04	-0.519 *	2.90E-17	2.579	1.80E-11	-	-
P13686	ACP5	0.093	1.70E-01	-0.248 *	1.00E-04	3.817	4.40E-22	-	-
Q9BUE0	MED18	0.114	9.00E-02	-0.261 *	1.10E-04	2.197	1.20E-08	-	-
P14868	DARS1	0.11	1.10E-01	-0.309 *	2.70E-06	1.491	1.00E-04	-	-
Q9P2X3	IMPACT	0.035	7.10E-01	-0.323 *	3.40E-07	1.514	1.10E-04	-	-
Q15366	PCBP2	0.109	1.10E-01	-0.398 *	6.30E-10	1.46	1.10E-04	-	-
Q9HB71	CACYBP	0.209 *	1.70E-04	-0.392 *	2.90E-10	2.664	3.70E-12	-	-
Q8TF64	GIPC3	0.207 *	1.70E-04	-0.480 *	3.50E-15	1.835	2.30E-06	-	-
O95497	VNN1	-0.115	7.90E-02	-0.254 *	1.20E-04	3.088	3.90E-16	-	-

P04406	GAPDH	-0.213 *	1.20E-04	-0.08	3.30E-01	2.209	1.20E-08	-	-
Q92835	INPP5D	0.068	3.80E-01	-0.251 *	1.20E-04	3.124	7.30E-17	-	-
P54284	CACNB3	0.082	2.70E-01	-0.460 *	7.40E-13	1.45	1.40E-04	-	-
P55273	CDKN2D	0.210 *	2.40E-04	-0.356 *	2.80E-08	1.8	2.40E-06	-	-
P17568	NDUFB7	0.086	2.40E-01	-0.253 *	1.60E-04	1.803	1.90E-06	-	-
Q9H4A9	DPEP2	-0.275 *	3.80E-08	0.574 *	3.60E-23	-1.504	2.70E-04	-	-
Q13043	STK4	0.207 *	2.60E-04	-0.374 *	3.50E-09	1.721	6.80E-06	-	-
Q14203	DCTN1	0.041	6.50E-01	-0.250 *	1.80E-04	2.444	1.10E-10	-	-
Q5JTD0	TJAP1	0.205 *	2.80E-04	-0.420 *	2.60E-11	2.855	4.30E-14	-	-
P01034	CST3	-0.188 *	2.80E-04	0.045	6.00E-01	2.333	2.40E-07	1.4	9.30E-06
P02749	APOH	0.079	2.80E-01	0.244 *	2.10E-04	2.124	5.00E-08	-	-
O60907	TBL1X	0.103	1.40E-01	-0.300 *	5.30E-06	1.448	2.00E-04	-	-
Q07011	TNFRSF9	-0.247 *	2.60E-06	0.048	5.90E-01	-	-	1.3	2.20E-04
O95841	ANGPTL1	0.05	5.40E-01	-0.360 *	2.90E-09	1.809	5.50E-06	1.3	3.30E-04
Q9UJU6	DBNL	0.202 *	3.30E-04	-0.416 *	3.70E-11	2.798	1.60E-13	-	-
O15169	AXIN1	0.203 *	3.30E-04	-0.406 *	1.30E-10	2.715	7.40E-13	-	-
Q9Y4D1	DAAM1	0.198 *	3.30E-04	-0.360 *	4.80E-09	2.79	7.30E-13	-	-
Q9NUY8	TBC1D23	0.212 *	2.00E-04	-0.255 *	1.20E-04	1.683	1.50E-05	-	-
Q07654	TFF3	-0.257 *	6.40E-07	0.052	5.50E-01	-	-	1.3	2.20E-04
Q6IBS0	TWF2	0.107	1.20E-01	-0.263 *	8.30E-05	1.452	1.40E-04	-	-
P43121	MCAM	-0.048	5.40E-01	-0.227 *	2.30E-04	3.128	8.00E-14	-	-
O76061	STC2	-0.096	1.50E-01	-0.238 *	2.30E-04	2.742	3.50E-12	-	-
O76074	PDE5A	0.198 *	3.50E-04	-0.584 *	8.80E-22	2.364	7.10E-10	-	-
O95544	NADK	0.209 *	2.30E-04	-0.006	9.60E-01	5.135	2.90E-43	-	-
P00995	SPINK1	-0.202 *	2.30E-04	-0.019	8.60E-01	-	-	1.4	1.50E-06
Q12778	FOXO1	0.202 *	3.60E-04	-0.376 *	3.20E-09	1.816	1.90E-06	-	-
P78333	GPC5	-0.003	9.80E-01	-0.266 *	4.60E-05	1.48	1.90E-04	-	-
Q92888	ARHGEF1	0.197 *	3.60E-04	-0.475 *	7.10E-15	3.1	1.80E-16	-	-
P14543	NID1	0.02	8.50E-01	-0.301 *	1.50E-06	1.555	1.40E-04	1.3	2.20E-04
P30533	LRPAP1	-0.026	7.90E-01	-0.242 *	2.50E-04	2.149	3.90E-08	-	-

O43312	MTSS1	0.116	8.10E-02	-0.377 *	6.00E-09	1.4	2.50E-04	-	-
P01303	NPY	0.01	9.30E-01	-0.244 *	2.70E-04	4.016	9.80E-27	-	-
Q6FI81	CIAPIN1	0.026	8.00E-01	-0.242 *	2.70E-04	2.614	7.90E-12	-	-
Q92583	CCL17	0.041	6.30E-01	-0.290 *	1.80E-06	-	-	1.3	2.70E-04
Q14767	LTBP2	-0.073	2.10E-01	-0.204 *	1.50E-04	2.599	2.80E-07	1.4	2.90E-04
Q15166	PON3	0.069	3.80E-01	-0.432 *	1.00E-11	-1.39	3.00E-04	-	-
P28908	TNFRSF8	-0.202 *	4.00E-04	0.286 *	1.00E-05	-1.554	6.40E-05	-	-
Q8WWN9	IPCEF1	0.201 *	3.20E-04	-0.076	3.60E-01	3.2	3.70E-17	-	-
P06858	LPL	-0.021	8.30E-01	-0.340 *	9.00E-09	-1.516	3.30E-04	-	-
Q6GMV3	PTRHD1	0.077	3.00E-01	-0.239 *	3.30E-04	1.973	2.30E-07	-	-
Q9BU02	THTPA	0.017	8.80E-01	-0.255 *	1.60E-04	1.429	1.70E-04	-	-
Q9P0G3	KLK14	0.097	1.60E-01	0.341 *	6.40E-08	-1.413	3.30E-04	-	-
P00813	ADA	-0.223 *	5.40E-05	0.017	8.80E-01	1.437	2.90E-04	-	-
Q9Y662	HS3ST3B1	-0.106	1.20E-01	-0.241 *	3.70E-04	3.374	2.10E-19	-	-
P07741	APRT	0.002	9.90E-01	-0.239 *	3.70E-04	1.776	4.80E-06	-	-
Q8TEA8	DTD1	0.203 *	3.70E-04	-0.363 *	1.10E-08	1.428	2.10E-04	-	-
O43405	COCH	-0.201 *	2.60E-04	-0.017	8.70E-01	1.485	3.00E-04	1.4	1.70E-05
P46531	NOTCH1	0.088	2.20E-01	-0.237 *	4.00E-04	1.93	3.80E-07	-	-
O43561	LAT	0.11	7.90E-02	-0.419 *	3.00E-12	1.906	1.90E-06	1.3	6.00E-04
O60496	DOK2	0.194 *	6.30E-04	-0.460 *	1.60E-13	2.495	7.50E-11	-	-
P07947	YES1	0.197 *	6.40E-04	-0.457 *	6.10E-13	3.207	1.60E-17	-	-
P13236	CCL4	-0.01	9.30E-01	-0.237 *	4.40E-04	1.565	4.00E-05	-	-
P07492	GRP	0.022	8.30E-01	0.222 *	4.80E-04	2.011	4.60E-07	-	-
P29965	CD40LG	-0.207 *	5.40E-05	-0.211 *	5.70E-04	-	-	1.3	9.70E-05
P50502	ST13	-0.06	4.50E-01	-0.229 *	4.90E-04	1.961	4.20E-07	-	-
Q96AJ9	VTI1A	-0.244 *	1.20E-05	0.058	5.30E-01	1.36	4.80E-04	-	-
P63098	PPP3R1	0.052	5.40E-01	-0.230 *	5.00E-04	3.625	8.30E-21	-	-
P24001	IL32	-0.233 *	1.80E-05	0.052	5.70E-01	-1.397	4.90E-04	-	-
Q6UX71	PLXDC2	0.044	6.30E-01	-0.237 *	3.30E-04	1.451	2.10E-04	-	-
P78380	OLR1	-0.255 *	1.40E-06	0.242 *	1.40E-04	1.366	6.80E-04	-	-

Q08174	PCDH1	0.103	1.40E-01	-0.364 *	1.70E-08	1.349	5.50E-04	-	-
I3L3R5	CCER2	-0.200 *	4.10E-04	0.453 *	3.80E-13	-1.375	4.30E-04	-	-
O94907	DKK1	-0.011	9.20E-01	-0.337 *	1.30E-08	1.404	8.70E-04	1.4	1.70E-05
P80511	S100A12	-0.069	3.80E-01	0.229 *	6.00E-04	2.904	2.10E-14	-	-
P39905	GDNF	-0.002	9.90E-01	-0.228 *	6.10E-04	1.835	5.80E-06	-	-
Q9Y5C1	ANGPTL3	-0.091	1.40E-01	-0.218 *	2.50E-04	2.21	4.60E-07	1.3	6.70E-04
Q15399	TLR1	0.031	7.60E-01	-0.236 *	5.10E-04	1.489	1.10E-04	-	-
P50225	SULT1A1	0.189 *	9.50E-04	-0.493 *	1.60E-15	2.147	1.80E-08	-	-
P34949	MPI	-0.206 *	2.30E-04	0.079	3.40E-01	1.506	1.50E-04	1.3	5.90E-04
Q8NI17	IL31RA	0.116	7.60E-02	-0.256 *	1.00E-04	1.361	5.80E-04	-	-
O00308	WWP2	0.072	3.60E-01	-0.233 *	6.90E-04	4.198	3.90E-30	-	-
Q99988	GDF15	-0.179 *	1.60E-04	-0.072	3.00E-01	-	-	1.4	5.50E-04
O00292	LEFTY2	-0.014	8.90E-01	-0.198 *	6.90E-04	1.919	2.80E-05	-	-
Q12933	TRAF2	0.190 *	1.10E-03	-0.337 *	1.50E-07	3.012	1.10E-15	-	-
P10644	PRKAR1A	0.062	4.50E-01	-0.230 *	7.40E-04	2.267	1.80E-09	-	-
Q99447	PCYT2	0.003	9.80E-01	-0.231 *	7.50E-04	1.985	1.20E-07	-	-
Q9BXN1	ASPN	-0.072	3.30E-01	-0.532 *	1.60E-18	1.339	7.70E-04	-	-
Q9ULL4	PLXNB3	0.192 *	1.20E-03	-0.273 *	4.00E-05	3.364	8.00E-20	-	-
P09486	SPARC	0.06	4.30E-01	-0.385 *	1.50E-10	1.809	7.90E-06	1.3	1.20E-03
P06730	EIF4E	0.031	7.60E-01	-0.255 *	1.40E-04	1.328	6.90E-04	-	-
P05451	REG1A	-0.204 *	2.30E-04	0.114	1.30E-01	-	-	1.3	5.90E-04
P46379	BAG6	-0.054	5.20E-01	-0.226 *	8.30E-04	3.205	8.00E-17	-	-
P78325	ADAM8	-0.01	9.30E-01	-0.223 *	8.40E-04	2.315	1.90E-09	-	-
Q9UKW4	VAV3	0.190 *	1.30E-03	-0.289 *	1.20E-05	1.74	5.50E-06	-	-
Q6NUJ1	PSAPL1	-0.07	3.10E-01	0.974 *	8.40E-66	-1.351	8.60E-04	-	-
P22692	IGFBP4	-0.170 *	1.30E-03	-0.028	7.60E-01	3.362	6.70E-14	1.4	1.10E-05
P63172	DYNLT1	0.188 *	1.40E-03	-0.432 *	1.10E-11	2.712	2.60E-13	-	-
Q8IZF2	ADGRF5	0.014	9.00E-01	-0.295 *	2.60E-06	1.327	9.70E-04	-	-
Q9ULX7	CA14	0.021	8.40E-01	-0.266 *	1.90E-05	-1.362	9.50E-04	-	-
Q9UHP3	USP25	0.104	1.30E-01	-0.228 *	9.70E-04	1.764	2.70E-06	-	-

P55957	BID	-0.095	1.50E-01	0.324 *	1.90E-07	1.3	1.00E-03	-	-
P52798	EFNA4	-0.190 *	5.50E-04	0.095	2.10E-01	1.707	6.60E-05	1.3	9.40E-04
P01137	TGFB1	-0.015	8.90E-01	-0.230 *	3.90E-04	3.188	1.30E-15	1.3	1.20E-03
P12111	COL6A3	-0.264 *	3.90E-07	0.026	8.00E-01	1.805	1.50E-05	1.3	1.60E-03
Q99704	DOK1	0.182 *	1.60E-03	-0.499 *	1.00E-15	1.851	1.60E-06	-	-
Q9NP84	TNFRSF12A	-0.180 *	8.20E-04	-0.091	2.30E-01	3.546	1.20E-16	1.3	7.70E-04
Q9Y2Z0	SUGT1	0.051	5.70E-01	-0.256 *	1.70E-04	1.296	9.10E-04	-	-
Q96CN9	GCC1	0.183 *	1.60E-03	-0.408 *	9.60E-11	2.157	1.70E-08	-	-
P07358	C8B	0.066	4.10E-01	-0.219 *	1.60E-03	1.953	1.90E-07	1.3	5.40E-05
Q9H7M9	VSIR	0.04	6.60E-01	-0.223 *	1.10E-03	2.332	9.30E-10	-	-
Q9H171	ZBP1	0.1	1.40E-01	0.220 *	1.10E-03	1.86	1.30E-06	-	-
Q96DR5	BPIFA2	0.088	2.30E-01	-0.248 *	2.50E-04	-1.279	8.50E-04	-	-
Q9H3G5	CPVL	0.064	4.30E-01	-0.227 *	1.00E-03	1.5	7.40E-05	-	-
Q9HAT2	SIAE	0.017	8.70E-01	-0.216 *	1.10E-03	1.645	4.80E-05	-	-
Q9UHF1	EGFL7	-0.073	3.20E-01	-0.515 *	3.00E-17	1.944	1.80E-06	1.3	1.70E-03
P15085	CPA1	0.087	2.20E-01	-0.225 *	7.50E-04	1.377	3.90E-04	-	-
O75888	TNFSF13	-0.03	7.50E-01	-0.207 *	1.40E-03	1.582	2.10E-04	1.4	1.00E-04
P23588	EIF4B	0.036	7.00E-01	-0.219 *	1.20E-03	2.144	2.40E-08	-	-
Q5R372	RABGAP1L	0.02	8.50E-01	-0.230 *	7.50E-04	1.358	4.40E-04	-	-
Q5SW96	LDLRAP1	0.216 *	1.40E-04	-0.433 *	8.40E-12	1.216	1.60E-03	-	-
Q05516	ZBTB16	0.191 *	1.30E-03	-0.238 *	5.20E-04	1.95	2.80E-07	-	-
Q9H446	RWDD1	0.03	7.60E-01	-0.218 *	1.20E-03	2.182	1.20E-08	-	-
Q05193	DNM1	0.243 *	1.20E-05	-0.307 *	1.90E-06	1.212	1.80E-03	-	-
P50454	SERPINH1	0.113	8.80E-02	-0.312 *	1.40E-06	1.26	1.20E-03	-	-
Q8WUX2	CHAC2	-0.008	9.50E-01	-0.238 *	4.60E-04	1.316	7.90E-04	-	-
Q8NDB2	BANK1	0.173 *	1.90E-03	-0.513 *	1.10E-17	2.824	5.40E-13	-	-
Q8IY22	CMIP	0.211 *	2.30E-04	-0.366 *	1.10E-08	1.214	1.70E-03	-	-
P12318	FCGR2A	-0.187 *	8.20E-04	-0.008	9.50E-01	1.425	4.60E-04	-	-
P07911	UMOD	0.171 *	1.30E-03	0.017	8.70E-01	-3.398	8.10E-16	-	-
Q14118	DAG1	0.027	7.90E-01	-0.216 *	1.30E-03	3.566	6.00E-20	-	-

P21589	NT5E	-0.041	6.50E-01	-0.218 *	1.30E-03	2.719	6.00E-13	-	-
Q86SQ7	SDCCAG8	0.178 *	2.00E-03	-0.394 *	3.20E-10	2.067	7.30E-08	-	-
Q8N4C8	MINK1	0.181 *	2.00E-03	-0.321 *	6.80E-07	1.946	3.90E-07	-	-
O43570	CA12	0.066	4.10E-01	-0.226 *	9.30E-04	1.367	4.60E-04	-	-
O95388	CCN4	-0.097	1.40E-01	-0.309 *	7.50E-07	3.044	3.50E-14	1.3	2.10E-03
P98160	HSPG2	-0.255 *	6.70E-07	-0.065	4.30E-01	2.68	2.20E-10	1.3	2.10E-03
Q9H3R2	MUC13	-0.101	1.20E-01	-0.208 *	1.40E-03	2.037	2.80E-07	-	-
P55808	XG	-0.343 *	4.10E-18	0.167 *	1.40E-03	1.716	7.30E-04	-	-
O15013	ARHGEF10	0.073	3.50E-01	-0.315 *	1.70E-06	1.22	1.40E-03	-	-
Q96RD9	FCRL5	0.032	7.50E-01	-0.219 *	1.50E-03	1.692	7.90E-06	-	-
Q8IVG5	SAMD9L	0.188 *	1.50E-03	-0.088	3.00E-01	1.761	3.90E-06	-	-
Q9BU40	CHRDL1	0.011	9.10E-01	-0.333 *	3.10E-09	1.506	1.50E-03	-	-
Q96RT1	ERBIN	0.178 *	2.20E-03	-0.284 *	1.00E-05	2.29	2.80E-09	-	-
P49137	MAPKAPK2	0.121	7.20E-02	-0.235 *	6.40E-04	1.272	8.50E-04	-	-
Q674X7	KAZN	0.176 *	2.20E-03	-0.505 *	3.00E-16	1.978	2.70E-07	-	-
Q9Y624	F11R	0.017	8.80E-01	-0.225 *	5.80E-04	3.976	8.80E-24	1.3	1.70E-03
Q14112	NID2	-0.065	3.80E-01	-0.325 *	9.00E-08	-	-	1.3	1.60E-03
P62760	VSNL1	-0.032	7.30E-01	0.205 *	1.60E-03	2.064	3.20E-07	-	-
Q92890	UFD1	0.183 *	2.10E-03	-0.244 *	2.90E-04	2.718	2.80E-13	-	-
O43291	SPINT2	-0.118	6.40E-02	-0.360 *	8.70E-09	1.351	8.70E-04	1.3	1.60E-03
P01133	EGF	0.037	6.60E-01	-0.235 *	1.30E-04	1.819	9.30E-06	1.2	2.40E-03
P56192	MARS1	0.179 *	2.50E-03	-0.336 *	2.40E-07	2.041	6.70E-08	-	-
Q765P7	MTSS2	0.180 *	2.50E-03	-0.354 *	3.70E-08	1.707	5.60E-06	-	-
Q9NWZ3	IRAK4	0.179 *	2.50E-03	-0.445 *	3.30E-12	2.561	8.10E-12	-	-
Q9Y6A5	TACC3	0.176 *	2.60E-03	-0.349 *	4.30E-08	1.792	4.30E-06	-	-
Q9NRR1	CYTL1	-0.215 *	5.60E-05	0.201 *	1.90E-03	-1.404	6.20E-04	-	-
Q15025	TNIP1	0.172 *	2.60E-03	-0.403 *	7.20E-11	2.493	5.10E-11	-	-
Q10471	GALNT2	-0.04	6.70E-01	-0.216 *	1.80E-03	1.81	1.80E-06	-	-
Q6GTX8	LAIR1	-0.164 *	2.60E-03	-0.005	9.70E-01	1.908	1.30E-05	1.4	5.40E-05
Q8NFL0	B3GNT7	-0.032	7.40E-01	-0.210 *	1.80E-03	1.812	5.20E-06	-	-

P07306	ASGR1	0.015	9.00E-01	-0.207 *	2.60E-03	4.286	9.50E-29	1.3	8.80E-05
P15848	ARSB	0.027	7.90E-01	-0.213 *	1.80E-03	2.113	3.00E-08	-	-
P09341	CXCL1	0.113	8.70E-02	-0.209 *	1.90E-03	1.459	2.00E-04	1.3	6.20E-04
Q9H4F8	SMOC1	0.037	6.80E-01	-0.297 *	1.50E-06	4.434	7.30E-28	1.3	2.80E-03
O43776	NARS1	0.178 *	2.80E-03	-0.409 *	1.40E-10	1.798	1.60E-06	-	-
P01241	GH1	0.174 *	1.90E-03	-0.076	3.40E-01	-2.186	5.90E-08	-	-
P62166	NCS1	-0.169 *	2.40E-03	-0.224 *	3.80E-04	3.632	2.90E-18	-	-
Q92520	FAM3C	-0.177 *	9.50E-04	-0.192 *	2.30E-03	2.854	4.30E-11	1.3	6.00E-04
Q6UWP8	SBSN	0.024	8.10E-01	0.635 *	4.80E-26	-1.223	1.90E-03	-	-
P40225	THPO	0.07	3.40E-01	-0.286 *	4.50E-06	1.685	4.70E-05	1.3	2.90E-03
P14174	MIF	0.034	7.30E-01	-0.210 *	2.00E-03	2.064	6.80E-08	-	-
Q6GQQ9	OTUD7B	-0.029	7.80E-01	-0.214 *	1.90E-03	1.506	9.30E-05	-	-
Q5T5Y3	CAMSAP1	0.178 *	2.50E-03	-0.380 *	2.40E-09	1.341	5.50E-04	-	-
Q9Y6K9	IKBKG	0.174 *	3.10E-03	-0.427 *	1.30E-11	3.128	6.90E-17	-	-
P20472	PVALB	0.183 *	1.00E-03	-0.423 *	4.20E-12	2.046	2.50E-07	1.2	3.20E-03
P05121	SERPINE1	0.061	4.00E-01	-0.269 *	6.50E-06	2.666	8.40E-11	1.3	3.20E-03
P43489	TNFRSF4	-0.176 *	2.20E-03	-0.052	5.70E-01	-	-	1.4	2.00E-05
O00175	CCL24	-0.12	6.50E-02	-0.282 *	1.60E-05	1.212	2.20E-03	-	-
O75077	ADAM23	-0.042	6.50E-01	-0.210 *	2.30E-03	2.265	2.90E-09	-	-
P07093	SERPINE2	-0.008	9.50E-01	-0.336 *	5.60E-08	1.224	2.30E-03	-	-
P36959	GMPR	-0.028	7.80E-01	-0.206 *	2.30E-03	2.298	2.50E-09	-	-
P30530	AXL	-0.168 *	2.20E-03	0.09	2.30E-01	1.663	7.60E-05	-	-
Q0ZGT2	NEXN	0.188 *	1.30E-03	-0.250 *	1.70E-04	1.202	1.90E-03	-	-
Q6UWW8	CES3	-0.016	8.90E-01	0.210 *	2.30E-03	1.869	1.30E-06	-	-
P78552	IL13RA1	-0.021	8.50E-01	-0.211 *	2.30E-03	3.414	3.60E-20	-	-
Q5JS37	NHLRC3	0.169 *	3.50E-03	-0.607 *	2.40E-23	3.776	4.80E-23	-	-
P21964	COMT	0.053	5.40E-01	-0.210 *	2.30E-03	2.444	2.00E-10	-	-
O95817	BAG3	-0.268 *	1.60E-06	0.204 *	3.50E-03	2.018	8.70E-08	-	-
P01298	PPY	0	1.00E+00	-0.185 *	2.40E-03	2.052	2.20E-06	-	-
P04080	CSTB	-0.201 *	7.70E-05	0.003	9.80E-01	1.351	3.10E-03	1.3	3.80E-04

P35579	MYH9	0.169 *	3.60E-03	-0.358 *	9.30E-09	2.987	4.80E-15	-	-
P52943	CRIP2	0.168 *	3.60E-03	-0.349 *	1.70E-08	5.427	1.40E-42	-	-
Q03405	PLAUR	-0.167 *	2.40E-03	0	1.00E+00	-	-	1.4	8.10E-06
P50579	METAP2	-0.015	8.90E-01	-0.209 *	2.50E-03	3.228	4.70E-18	-	-
P35318	ADM	-0.158 *	3.70E-03	-0.087	2.40E-01	3.043	8.60E-12	1.5	5.80E-06
P25774	CTSS	-0.298 *	3.20E-08	0.197 *	3.80E-03	1.709	9.20E-06	-	-
Q96PL5	ERMAP	-0.242 *	5.60E-06	0.287 *	5.20E-06	1.186	4.00E-03	-	-
Q6UWV6	ENPP7	-0.108	1.20E-01	-0.208 *	2.70E-03	1.954	2.60E-07	-	-
Q9ULI3	HEG1	0.052	5.40E-01	-0.204 *	2.70E-03	5.098	2.60E-41	-	-
P08134	RHOC	0.164 *	4.20E-03	-0.457 *	4.00E-14	2.35	2.90E-09	-	-
O14974	PPP1R12A	0.170 *	4.20E-03	-0.293 *	7.80E-06	3.025	9.10E-16	-	-
Q9UMR7	CLEC4A	0.168 *	4.30E-03	-0.449 *	5.80E-13	3.864	7.70E-25	-	-
P13725	OSM	-0.232 *	2.50E-05	0.112	1.40E-01	1.178	2.80E-03	-	-
P57087	JAM2	-0.265 *	2.30E-07	0.131	5.90E-02	-	-	1.3	2.90E-03
P14091	CTSE	-0.088	1.90E-01	-0.269 *	1.90E-05	1.985	3.00E-07	0.8	4.30E-03
P41208	CETN2	-0.013	9.10E-01	-0.222 *	1.20E-03	1.22	1.70E-03	-	-
Q9UKY0	PRND	-0.181 *	2.60E-03	-0.005	9.70E-01	-1.38	3.00E-04	-	-
O75354	ENTPD6	0.077	3.00E-01	-0.203 *	3.00E-03	2.198	1.90E-08	-	-
O75711	SCRG1	-0.203 *	8.70E-05	-0.115	9.80E-02	1.323	2.90E-03	-	-
P35625	TIMP3	0.01	9.30E-01	-0.290 *	4.30E-06	1.671	2.40E-05	1.2	4.60E-03
O60763	USO1	0.083	2.80E-01	-0.209 *	3.00E-03	1.529	6.60E-05	-	-
Q8IU57	IFNLR1	0.029	7.80E-01	-0.206 *	3.10E-03	3.155	1.40E-17	-	-
P04085	PDGFA	0.034	6.90E-01	-0.403 *	5.80E-12	1.299	1.90E-03	1.3	2.80E-03
Q96CG8	CTHRC1	0.007	9.50E-01	-0.186 *	3.10E-03	3.213	4.50E-14	-	-
Q6ZRY4	RBPMS2	0.165 *	4.70E-03	-0.420 *	1.80E-11	2.312	2.20E-09	-	-
P31949	S100A11	-0.065	4.00E-01	0.199 *	3.20E-03	2.98	8.90E-15	-	-
P06733	ENO1	0.170 *	4.80E-03	-0.358 *	3.50E-08	3.546	5.10E-22	-	-
O00559	EBAG9	0.117	7.90E-02	-0.204 *	3.20E-03	1.986	2.00E-07	-	-
P07948	LYN	0.126	5.50E-02	-0.205 *	3.30E-03	2.48	4.70E-11	-	-
P00750	PLAT	0.166 *	3.30E-03	-0.118	1.00E-01	5.403	2.10E-43	-	-

O95630	STAMBP	-0.06	4.70E-01	-0.202 *	3.30E-03	2.511	5.70E-11	-	-
Q9H6B4	CLMP	-0.168 *	2.60E-03	-0.085	2.70E-01	2.355	2.00E-08	1.3	2.40E-03
Q6P589	TNFAIP8L2	0.176 *	3.10E-03	-0.213 *	1.90E-03	1.934	3.20E-07	-	-
P19438	TNFRSF1A	-0.160 *	4.20E-03	0.03	7.50E-01	2.046	2.40E-06	1.3	8.10E-04
P55145	MANF	0.162 *	5.10E-03	-0.456 *	7.20E-14	2.17	2.90E-08	-	-
Q8NBP7	PCSK9	-0.009	9.40E-01	-0.232 *	3.70E-04	3.272	1.30E-17	1.2	4.90E-03
P50995	ANXA11	0.172 *	3.50E-03	-0.103	1.90E-01	2.275	2.40E-09	-	-
Q96AP7	ESAM	-0.022	8.20E-01	-0.182 *	5.00E-03	1.977	2.20E-06	1.3	3.50E-04
Q9BXJ1	C1QTNF1	0.036	7.10E-01	-0.202 *	3.60E-03	2.098	3.90E-08	-	-
	NTproBNP	-0.154 *	5.50E-03	-0.023	8.20E-01	3.152	1.60E-13	1.8	4.30E-18
Q86VP1	TAX1BP1	0.126	5.30E-02	-0.202 *	3.70E-03	2.307	9.40E-10	-	-
P31751	AKT2	0.165 *	5.60E-03	-0.471 *	6.20E-14	1.845	1.10E-06	-	-
O75154	RAB11FIP3	0.160 *	5.60E-03	-0.501 *	1.90E-16	1.752	7.60E-06	-	-
O15240	VGF	-0.171 *	3.70E-03	0.031	7.60E-01	-1.859	1.40E-06	-	-
Q9UJ70	NAGK	0.045	6.20E-01	-0.199 *	3.80E-03	2.392	3.40E-10	-	-
Q9UKP3	ITGB1BP2	0.231 *	1.60E-05	-0.540 *	2.70E-19	2.212	2.30E-08	1.2	7.50E-03
P29692	EEF1D	0.165 *	5.70E-03	-0.387 *	1.60E-09	2.998	6.10E-16	-	-
Q92558	WASF1	0.085	2.50E-01	-0.327 *	8.20E-07	1.138	3.80E-03	-	-
Q6PJW8	CNST	0.162 *	5.80E-03	-0.507 *	3.60E-16	2.726	6.30E-13	-	-
Q14162	SCARF1	-0.096	1.50E-01	-0.224 *	7.00E-04	2.323	3.00E-09	1.2	5.10E-03
O14763	TNFRSF10B	-0.207 *	4.60E-05	-0.031	7.30E-01	1.326	5.70E-03	1.4	8.10E-05
Q93052	LPP	0.106	1.20E-01	-0.214 *	2.00E-03	1.199	1.90E-03	-	-
Q9H4D0	CLSTN2	-0.113	8.50E-02	-0.203 *	2.80E-03	1.3	1.10E-03	-	-
Q9NPY3	CD93	-0.021	8.40E-01	-0.214 *	9.30E-04	1.246	3.00E-03	-	-
P01589	IL2RA	-0.079	2.80E-01	0.198 *	3.90E-03	-	-	1.3	5.90E-05
P30838	ALDH3A1	0.104	1.20E-01	-0.250 *	1.40E-04	1.166	3.80E-03	-	-
Q76M96	CCDC80	0.102	7.40E-02	-0.173 *	4.00E-03	2.037	1.30E-05	-	-
Q9C035	TRIM5	0.166 *	6.00E-03	-0.265 *	8.30E-05	1.917	3.90E-07	-	-
Q14011	CIRBP	0.163 *	6.10E-03	-0.393 *	4.70E-10	1.755	4.00E-06	-	-
Q92686	NRGN	0.187 *	1.30E-03	-0.391 *	5.30E-10	1.106	4.90E-03	-	-

O00273	DFFA	0.057	5.00E-01	-0.198 *	4.10E-03	2.812	5.70E-14	-	-
P49767	VEGFC	-0.029	7.60E-01	-0.382 *	2.70E-10	1.678	3.70E-05	1.2	6.20E-03
Q99538	LGMN	0.170 *	4.20E-03	-0.118	1.30E-01	1.728	6.00E-06	-	-
P05305	EDN1	-0.163 *	5.40E-03	-0.062	4.80E-01	1.394	6.10E-04	1.3	2.70E-04
Q5KU26	COLEC12	-0.237 *	1.60E-06	-0.011	9.20E-01	1.29	5.70E-03	1.3	6.60E-04
Q9NS68	TNFRSF19	-0.156 *	4.20E-03	0.006	9.50E-01	-	-	1.4	9.50E-05
Q9BTE6	AARSD1	-0.06	4.40E-01	-0.189 *	4.30E-03	1.835	5.30E-06	-	-
P07949	RET	0.156 *	6.50E-03	-0.675 *	6.40E-30	3.581	3.90E-20	-	-
Q03426	MVK	0.02	8.50E-01	-0.198 *	4.30E-03	3.209	1.80E-17	-	-
Q9UQQ2	SH2B3	0.164 *	6.50E-03	-0.346 *	8.40E-08	1.627	2.70E-05	-	-
O95393	BMP10	-0.027	7.80E-01	-0.204 *	1.70E-03	1.257	2.70E-03	-	-
Q02880	TOP2B	0.06	4.70E-01	-0.200 *	4.40E-03	3.098	1.20E-16	-	-
P80370	DLK1	-0.220 *	4.00E-05	-0.182 *	5.90E-03	1.409	6.70E-04	-	-
P10646	TFPI	0.015	8.90E-01	-0.263 *	5.70E-05	2.843	1.60E-13	1.2	6.60E-03
Q9NPJ3	ACOT13	0.165 *	6.50E-03	-0.316 *	1.50E-06	1.413	1.80E-04	-	-
Q9BQS7	HEPH	0.047	6.00E-01	-0.196 *	4.50E-03	2.438	1.20E-10	-	-
P22894	MMP8	-0.320 *	1.60E-09	0.205 *	2.30E-03	1.117	4.40E-03	-	-
Q9Y240	CLEC11A	0.045	6.20E-01	-0.199 *	4.50E-03	1.583	3.60E-05	-	-
O14558	HSPB6	-0.252 *	2.20E-07	-0.232 *	7.90E-05	3.606	9.90E-16	1.3	9.00E-03
Q9Y2X7	GIT1	0.162 *	6.90E-03	-0.450 *	1.30E-12	1.685	1.30E-05	-	-
Q05084	ICA1	0.102	1.50E-01	-0.215 *	2.30E-03	1.178	2.30E-03	-	-
O00626	CCL22	-0.043	6.30E-01	0.278 *	1.90E-05	1.124	4.60E-03	-	-
Q7Z569	BRAP	0.159 *	7.00E-03	-0.527 *	2.70E-17	2.386	2.90E-10	-	-
Q15848	ADIPOQ	0.001	9.90E-01	-0.186 *	4.70E-03	-2.02	4.80E-07	-	-
O60502	OGA	0.163 *	7.10E-03	-0.287 *	1.70E-05	2.347	2.50E-10	-	-
Q7Z5A7	TAFA5	-0.304 *	3.20E-09	0.210 *	1.10E-03	-	-	1.3	6.00E-03
Q09666	AHNAK	-0.067	3.90E-01	-0.193 *	4.70E-03	1.616	3.70E-05	-	-
P16109	SELP	0.092	1.60E-01	-0.381 *	2.70E-10	3.457	2.80E-18	1.2	7.30E-03
Q86SJ2	AMIGO2	0.071	2.70E-01	-0.168 *	5.00E-03	3.952	1.30E-18	-	-
P29536	LMOD1	-0.038	6.50E-01	-0.222 *	2.70E-04	3.37	6.00E-15	1.2	7.20E-03

Q96SM3	CPXM1	-0.003	9.80E-01	-0.401 *	3.60E-11	1.491	2.10E-04	1.2	7.30E-03
O75940	SMNDC1	-0.014	9.00E-01	0.190 *	4.20E-03	1.303	8.70E-04	-	-
P48745	CCN3	-0.151 *	6.40E-03	-0.203 *	1.20E-03	2.685	2.20E-10	-	-
O14917	PCDH17	0.108	8.80E-02	-0.199 *	2.30E-03	1.241	2.80E-03	-	-
Q9UEW3	MARCO	-0.148 *	7.40E-03	-0.226 *	2.40E-04	1.771	3.40E-05	-	-
Q07325	CXCL9	-0.222 *	3.70E-05	0.193 *	3.40E-03	-1.134	6.80E-03	1.3	5.60E-06
P27348	YWHAQ	0.161 *	7.80E-03	-0.359 *	2.30E-08	2.019	8.80E-08	-	-
Q9BRF8	CPPED1	-0.165 *	5.20E-03	-0.039	6.90E-01	2.061	8.40E-08	-	-
P01127	PDGFB	-0.002	9.80E-01	-0.389 *	1.80E-11	1.15	7.80E-03	1.3	2.10E-04
O60890	OPHN1	0.158 *	7.80E-03	-0.466 *	7.20E-14	1.432	2.20E-04	-	-
P62330	ARF6	0.073	3.50E-01	-0.194 *	5.40E-03	2.378	2.40E-10	-	-
P10144	GZMB	-0.272 *	4.90E-07	0.062	4.80E-01	1.1	5.50E-03	-	-
Q9NRJ3	CCL28	0.023	8.20E-01	-0.186 *	5.40E-03	-	-	1.3	5.00E-05
Q12986	NFX1	0.082	2.80E-01	-0.238 *	5.50E-04	1.084	4.90E-03	-	-
Q16543	CDC37	0.161 *	8.40E-03	-0.289 *	1.30E-05	2.137	1.20E-08	-	-
P05413	FABP3	-0.234 *	5.30E-06	-0.013	9.00E-01	1.626	1.60E-04	1.2	8.20E-03
Q15389	ANGPT1	-0.042	6.10E-01	-0.438 *	2.60E-14	1.157	7.80E-03	1.3	6.80E-04
Q9Y5Q6	INSL5	-0.170 *	4.70E-03	0.066	4.60E-01	1.285	9.60E-04	-	-
P05067	APP	-0.032	7.30E-01	-0.352 *	5.70E-09	1.1	8.00E-03	1.3	5.70E-04
P40933	IL15	0.186 *	1.30E-03	-0.185 *	6.90E-03	-	-	1.3	4.40E-04
O75569	PRKRA	0.086	2.40E-01	-0.193 *	5.80E-03	2.671	1.50E-12	-	-
Q9NQ30	ESM1	-0.159 *	5.80E-03	0.067	4.20E-01	-1.854	3.60E-06	-	-
P24592	IGFBP6	-0.184 *	3.00E-04	0.014	8.90E-01	1.701	1.70E-04	1.2	8.30E-03
Q93015	NAA80	0.05	5.80E-01	-0.226 *	1.20E-03	1.093	4.70E-03	-	-
P06744	GPI	0.168 *	5.90E-03	0.061	5.10E-01	2.052	3.50E-08	-	-
O00300	TNFRSF11B	0.01	9.20E-01	-0.165 *	5.90E-03	4.022	5.70E-18	-	-
Q9UDT6	CLIP2	0.194 *	6.10E-04	-0.391 *	4.80E-10	1.604	3.80E-05	1.2	1.10E-02
Q9NR12	PDLIM7	0.154 *	9.00E-03	-0.418 *	1.60E-11	2.839	1.90E-13	-	-
P12081	HARS1	0.159 *	9.10E-03	-0.304 *	3.40E-06	2.253	2.20E-09	-	-
P53367	ARFIP1	0.047	6.10E-01	-0.249 *	2.70E-04	1.062	5.80E-03	-	-

Q8N6M0	OTUD6B	0.075	3.40E-01	-0.216 *	2.10E-03	1.1	4.00E-03	-	-
P51888	PRELP	-0.148 *	6.10E-03	-0.047	5.80E-01	1.713	1.50E-04	-	-
Q8IXM2	BAP18	0.055	5.20E-01	-0.188 *	6.20E-03	2.432	8.50E-11	-	-
P10747	CD28	-0.167 *	4.60E-03	0.048	6.10E-01	1.282	1.60E-03	-	-
Q6BAA4	FCRLB	-0.106	1.20E-01	-0.222 *	9.60E-04	2.099	9.40E-08	1.2	8.40E-03
Q9H6S1	AZI2	0.109	1.10E-01	-0.344 *	1.40E-07	1.051	6.30E-03	-	-
O95445	APOM	0.016	8.90E-01	-0.191 *	6.30E-03	1.897	5.90E-07	-	-
Q14956	GPNMB	-0.089	1.90E-01	-0.174 *	9.40E-03	2.145	1.90E-07	1.4	1.10E-04
Q9BUD6	SPON2	-0.159 *	3.10E-03	-0.335 *	9.20E-09	2.597	4.10E-10	1.2	9.70E-03
P32970	CD70	-0.072	3.10E-01	0.239 *	1.20E-04	-	-	1.2	6.30E-03
Q9NP79	VTA1	-0.039	6.70E-01	-0.187 *	6.50E-03	2.36	5.60E-10	-	-
Q14005	IL16	-0.038	6.70E-01	0.187 *	6.50E-03	4.125	1.30E-27	-	-
P22676	CALB2	-0.155 *	7.00E-03	0.293 *	2.60E-06	1.253	2.80E-03	-	-
P37235	HPCAL1	0.165 *	6.10E-03	-0.352 *	4.20E-08	1.913	4.30E-07	1.2	7.20E-03
O14791	APOL1	0.163 *	6.60E-03	0.095	2.40E-01	1.941	4.00E-07	-	-
Q8TE58	ADAMTS15	-0.154 *	1.00E-02	-0.347 *	3.80E-08	2.81	1.40E-13	-	-
Q5GAN6	RNASE10	-0.170 *	4.30E-03	-0.008	9.40E-01	-	-	1.2	2.40E-03
Q15797	SMAD1	0.103	1.40E-01	-0.191 *	6.80E-03	1.788	3.50E-06	-	-
Q8NEZ2	VPS37A	0.158 *	1.00E-02	-0.262 *	9.80E-05	1.842	1.20E-06	-	-
Q00722	PLCB2	0.160 *	8.60E-03	-0.234 *	5.70E-04	1.246	1.30E-03	-	-
O15018	PDZD2	0.005	9.70E-01	-0.217 *	1.90E-03	1.092	5.10E-03	-	-
O43715	TRIAP1	-0.159 *	6.40E-03	0.007	9.50E-01	1.2	4.30E-03	1.4	2.00E-06
Q03167	TGFBR3	-0.106	1.10E-01	-0.275 *	2.20E-05	1.093	7.20E-03	-	-
Q14393	GAS6	-0.094	1.40E-01	-0.374 *	3.60E-10	1.152	7.20E-03	-	-
Q96SB3	PPP1R9B	0.154 *	1.00E-02	-0.230 *	5.10E-04	2.432	2.60E-10	-	-
P20774	OGN	-0.165 *	1.40E-03	-0.04	6.40E-01	1.407	2.50E-03	1.3	7.20E-03
P38936	CDKN1A	0.09	2.00E-01	-0.287 *	9.70E-06	2.331	1.30E-09	1.2	1.10E-02
P31350	RRM2	-0.031	7.70E-01	-0.191 *	7.70E-03	1.847	1.20E-06	-	-
Q13275	SEMA3F	-0.016	8.90E-01	-0.185 *	7.70E-03	3.651	5.20E-22	-	-
Q9BRK3	MXRA8	-0.016	8.70E-01	-0.201 *	1.40E-03	-2.128	4.00E-07	0.8	1.00E-02

Q9NWQ8	PAG1	-0.01	9.20E-01	0.157 *	5.40E-03	1.341	2.30E-03	-	-
O60941	DTNB	-0.154 *	6.60E-03	-0.122	8.60E-02	2.371	1.80E-08	1.3	5.10E-03
O14773	TPP1	0.104	1.30E-01	-0.186 *	7.80E-03	2.467	5.90E-11	-	-
Q99075	HBEGF	-0.003	9.80E-01	-0.255 *	2.10E-05	2.42	6.30E-09	1.2	1.20E-02
Q14914	PTGR1	-0.123	5.70E-02	-0.185 *	8.10E-03	1.612	2.60E-05	-	-
Q5VTT5	MYOM3	-0.305 *	8.00E-09	-0.14	5.10E-02	1.082	8.10E-03	-	-
P03956	MMP1	-0.027	7.80E-01	-0.527 *	1.70E-17	1.019	1.20E-02	1.3	5.80E-04
O60234	GMFG	0.084	2.60E-01	-0.190 *	6.90E-03	1.237	1.30E-03	-	-
P58499	FAM3B	-0.082	2.20E-01	-0.227 *	2.50E-04	1.178	5.60E-03	1.2	6.50E-03
P39900	MMP12	-0.284 *	1.50E-09	0.163 *	7.40E-03	-	-	1.3	5.20E-03
O60575	SPINK4	-0.183 *	1.30E-03	-0.04	6.70E-01	-	-	1.2	7.20E-03
P34896	SHMT1	0.037	7.00E-01	-0.188 *	8.40E-03	2.674	4.40E-13	-	-
P45984	MAPK9	-0.09	2.00E-01	0.180 *	8.70E-03	2.657	1.50E-11	-	-
Q9BQB4	SOST	-0.150 *	9.00E-03	-0.006	9.60E-01	2.245	3.30E-08	-	-
P02775	PPBP	0.017	8.60E-01	-0.403 *	1.10E-11	1.124	7.20E-03	1.2	6.30E-03
P42830	CXCL5	0.180 *	6.90E-04	-0.475 *	1.80E-16	1.143	7.20E-03	1.2	1.00E-02
P48061	CXCL12	0.11	1.00E-01	-0.186 *	8.30E-03	1.298	8.60E-04	-	-
P19876	CXCL3	0.183 *	6.40E-04	-0.401 *	1.10E-11	1.124	7.40E-03	1.2	1.00E-02
Q14790	CASP8	0.065	3.90E-01	0.177 *	9.10E-03	2.259	1.00E-08	-	-
O75843	AP1G2	0.106	1.30E-01	-0.187 *	9.20E-03	1.574	3.30E-05	-	-
P25116	F2R	0.076	2.80E-01	-0.240 *	1.40E-04	3.014	6.50E-14	1.2	1.40E-02
P29474	NOS3	0.032	7.50E-01	-0.185 *	9.50E-03	3.186	5.10E-17	-	-
Q9Y251	HPSE	0.024	8.10E-01	-0.324 *	2.00E-07	1.009	1.40E-02	1.3	5.70E-04
Q8N114	SHISA5	-0.150 *	7.00E-03	0.131	5.70E-02	-	-	1.3	2.80E-03
Q6WN34	CHRDL2	-0.112	9.80E-02	-0.183 *	9.80E-03	1.871	7.40E-07	-	-
Q9NRG1	PRTFDC1	0.153 *	1.20E-02	-0.376 *	3.50E-09	1.201	2.50E-03	-	-
P14210	HGF	-0.161 *	5.60E-03	-0.176 *	9.20E-03	3.678	2.30E-20	-	-
Q96KG7	MEGF10	-0.109	1.10E-01	-0.180 *	9.90E-03	1.747	5.90E-06	-	-
P13726	F3	-0.038	6.60E-01	-0.228 *	2.40E-04	1.596	2.00E-04	1.2	1.40E-02
P20333	TNFRSF1B	-0.172 *	2.30E-03	0.009	9.40E-01	-	-	1.2	7.80E-03

Q9Y3P8	SIT1	0.035	6.40E-01	0.182 *	1.10E-03	1.141	9.10E-03	-	-
P21741	MDK	-0.018	8.60E-01	-0.175 *	9.90E-03	-	-	1.3	2.90E-04
P07204	THBD	-0.014	9.00E-01	-0.175 *	1.00E-02	4.004	2.00E-24	-	-
P10147	CCL3	0.046	5.80E-01	-0.172 *	1.00E-02	2.264	3.60E-08	-	-
P56470	LGALS4	-0.266 *	2.50E-07	-0.101	1.70E-01	1.099	1.00E-02	-	-
P04066	FUCA1	-0.07	3.70E-01	-0.180 *	1.00E-02	2.046	8.60E-08	-	-
P08397	HMBS	-0.144 *	1.00E-02	0.08	3.00E-01	1.71	2.90E-05	-	-
P02654	APOC1	0.003	9.80E-01	-0.183 *	1.10E-02	1.751	3.30E-06	-	-
P06127	CD5	-0.091	1.80E-01	0.175 *	1.10E-02	2.284	5.40E-09	-	-
Q9UNK0	STX8	-0.369 *	3.10E-12	0.176 *	1.30E-02	-	-	1.2	3.40E-03
Q14508	WFDC2	-0.147 *	5.40E-03	-0.158 *	1.10E-02	-	-	1.7	3.90E-12
P47712	PLA2G4A	0.150 *	1.20E-02	-0.509 *	3.00E-16	3.448	6.70E-20	1.2	9.30E-03
Q9UK05	GDF2	0.031	7.40E-01	-0.171 *	1.10E-02	2.44	9.30E-10	-	-
P06748	NPM1	-0.04	6.50E-01	0.172 *	1.10E-02	1.545	8.00E-05	-	-
P35237	SERPINB6	0.002	9.80E-01	-0.171 *	1.10E-02	2.231	4.40E-08	-	-
P01033	TIMP1	-0.053	5.00E-01	-0.160 *	1.60E-02	2.497	3.90E-09	1.3	5.90E-04
Q9UBW5	BIN2	0.146 *	1.70E-02	-0.452 *	5.90E-13	2.224	7.70E-09	-	-
P41271	NBL1	-0.159 *	4.60E-03	-0.067	4.10E-01	-	-	1.2	6.50E-03
Q96A49	SYAP1	0.017	8.80E-01	-0.182 *	1.10E-02	1.468	1.10E-04	-	-
Q9HCN6	GP6	0.141 *	1.70E-02	-0.325 *	1.20E-07	3.247	7.90E-17	-	-
Q9UHX3	ADGRE2	0.141 *	1.70E-02	-0.435 *	6.20E-13	4.003	9.50E-25	-	-
Q9NR28	DIABLO	0.146 *	1.70E-02	-0.375 *	3.30E-09	2.155	1.90E-08	-	-
P50453	SERPINB9	0.096	1.70E-01	-0.417 *	4.70E-11	1.711	9.70E-06	1.2	1.70E-02
P28838	LAP3	0.163 *	8.80E-03	0.024	8.30E-01	1.16	2.60E-03	-	-
P00746	CFD	-0.157 *	8.20E-03	0.181 *	8.60E-03	1.132	5.60E-03	1.3	7.40E-04
Q53H82	LACTB2	0.019	8.60E-01	-0.265 *	7.40E-05	2.207	5.70E-09	1.2	1.70E-02
Q99683	MAP3K5	0.146 *	1.70E-02	-0.291 *	6.90E-06	1.578	6.50E-05	-	-
Q13137	CALCOCO2	0.148 *	1.70E-02	-0.297 *	7.30E-06	2.031	8.50E-08	-	-
P46937	YAP1	-0.200 *	7.70E-05	0.151 *	1.80E-02	2.045	4.20E-06	-	-
Q6QNY0	BLOC1S3	0.106	1.30E-01	-0.213 *	2.30E-03	1.003	9.60E-03	-	-

Q9UKU9	ANGPTL2	0.153 *	1.20E-02	-0.106	1.80E-01	2.888	2.50E-14	-	-
P29466	CASP1	-0.041	6.60E-01	0.203 *	3.30E-03	1.053	8.60E-03	-	-
P07237	P4HB	-0.015	8.90E-01	-0.280 *	9.60E-06	3.234	9.30E-17	1.2	1.80E-02
P23467	PTPRB	0.178 *	2.10E-03	-0.167 *	1.60E-02	6.348	6.70E-64	-	-
Q9NWM8	FKBP14	0.052	5.60E-01	-0.180 *	1.20E-02	1.448	1.40E-04	-	-
P24821	TNC	-0.05	5.60E-01	-0.176 *	1.20E-02	-	-	1.3	2.80E-05
P25445	FAS	-0.061	4.30E-01	-0.167 *	1.30E-02	2.387	7.20E-09	-	-
Q12765	SCRN1	0.053	5.40E-01	0.182 *	1.00E-02	1.187	2.40E-03	-	-
Q8N436	CPXM2	-0.11	9.90E-02	-0.175 *	1.30E-02	1.768	6.30E-06	-	-
Q14515	SPARCL1	0.078	3.10E-01	-0.216 *	1.70E-03	1.616	2.40E-05	1.2	1.70E-02
O94760	DDAH1	-0.151 *	1.20E-02	0.067	4.40E-01	1.411	4.70E-04	-	-
Q9H3U7	SMOC2	-0.109	7.70E-02	-0.158 *	1.90E-02	2.438	1.00E-08	1.3	4.00E-04
P40222	TXLNA	0.142 *	1.90E-02	-0.334 *	1.60E-07	2.991	5.70E-15	-	-
P30044	PRDX5	0.07	3.70E-01	-0.187 *	7.10E-03	2.978	5.70E-15	1.2	1.20E-02
Q96HC4	PDLIM5	0.144 *	2.00E-02	-0.449 *	8.90E-13	1.898	5.80E-07	-	-
Q9H777	ELAC1	0.145 *	2.00E-02	-0.446 *	2.10E-12	2.112	1.80E-08	-	-
O14841	OPLAH	0.081	2.90E-01	-0.183 *	1.10E-02	1.165	2.40E-03	-	-
O95467	GNAS	0.069	3.90E-01	-0.229 *	9.80E-04	0.973	1.20E-02	-	-
Q06141	REG3A	-0.141 *	1.60E-02	0.079	3.20E-01	-1.228	4.10E-03	1.4	2.00E-05
Q15633	TARBP2	0.115	9.10E-02	-0.178 *	1.40E-02	2.206	6.60E-09	-	-
P18510	IL1RN	-0.125	5.10E-02	0.173 *	1.40E-02	2.151	2.20E-08	-	-
Q8IWV2	CNTN4	-0.024	8.10E-01	-0.169 *	1.40E-02	1.758	1.00E-05	-	-
P19957	PI3	-0.224 *	1.70E-05	0.564 *	3.60E-22	-	-	1.2	2.00E-02
Q9NZN3	EHD3	0.236 *	1.00E-05	-0.527 *	2.80E-18	1.517	1.20E-04	1.2	2.70E-02
O75173	ADAMTS4	-0.024	8.20E-01	-0.177 *	1.10E-02	1.234	2.20E-03	-	-
Q9NYY1	IL20	0.028	7.90E-01	0.302 *	6.70E-06	-0.966	1.40E-02	-	-
P15090	FABP4	-0.151 *	3.60E-03	0.067	3.70E-01	4.039	1.60E-20	1.3	1.70E-02
Q8NBZ7	UXS1	-0.066	4.10E-01	-0.175 *	1.40E-02	1.545	7.50E-05	-	-
Q9BXN2	CLEC7A	-0.152 *	8.70E-03	-0.199 *	2.40E-03	-	-	1.2	1.00E-02
P54577	YARS1	0.145 *	2.10E-02	-0.355 *	5.00E-08	1.544	4.50E-05	-	-

P09382	LGALS1	-0.202 *	8.80E-05	-0.144 *	2.80E-02	2.703	1.80E-10	1.4	1.80E-04
Q9GZY6	LAT2	0.144 *	2.10E-02	-0.237 *	4.20E-04	3.401	4.60E-19	-	-
Q9BY76	ANGPTL4	-0.132 *	2.80E-02	-0.353 *	7.60E-09	1.938	2.50E-06	1.3	8.20E-04
P13987	CD59	-0.155 *	4.60E-03	0.096	1.90E-01	2.283	1.70E-07	1.2	1.70E-02
Q8WUF8	FAM172A	-0.003	9.80E-01	-0.182 *	1.10E-02	1.131	3.70E-03	-	-
P23743	DGKA	0.089	2.20E-01	-0.279 *	3.00E-05	0.966	1.50E-02	-	-
Q04760	GLO1	-0.215 *	5.00E-05	-0.154 *	2.20E-02	2.184	1.20E-07	-	-
Q9Y5L3	ENTPD2	0.076	3.30E-01	-0.176 *	1.50E-02	1.586	3.50E-05	-	-
Q6UX15	LAYN	-0.141 *	1.10E-02	-0.096	1.80E-01	-	-	1.3	4.60E-03
Q9NPG4	PCDH12	0.016	8.80E-01	-0.312 *	1.00E-06	-	-	0.8	1.50E-02
Q99685	MGLL	0.243 *	2.60E-06	-0.575 *	9.00E-23	3.469	7.00E-19	1.2	3.10E-02
P30039	PBLD	-0.06	4.70E-01	-0.174 *	1.60E-02	1.73	7.40E-06	-	-
Q9BYC5	FUT8	0.036	7.00E-01	-0.351 *	3.90E-08	1.671	1.60E-05	1.2	2.40E-02
O95644	NFATC1	0.140 *	2.40E-02	-0.395 *	4.50E-10	2.577	1.30E-11	-	-
Q8TDQ0	HAVCR2	-0.149 *	6.40E-03	0.005	9.70E-01	1.183	9.50E-03	-	-
O15354	GPR37	-0.147 *	1.60E-02	0.063	4.70E-01	3.222	3.80E-16	-	-
Q6UXG3	CD300LG	-0.105	1.10E-01	-0.199 *	2.70E-03	1.041	1.40E-02	-	-
P20849	COL9A1	0.027	7.90E-01	-0.167 *	1.60E-02	-	-	1.3	2.70E-04
P41236	PPP1R2	0.088	2.20E-01	-0.203 *	2.80E-03	2.754	5.40E-13	1.2	2.20E-02
P01275	GCG	-0.135 *	1.00E-02	0.048	5.50E-01	1.247	6.00E-03	-	-
Q96A25	TMEM106A	0.08	2.90E-01	-0.307 *	2.60E-06	0.942	1.70E-02	-	-
P05556	ITGB1	-0.138 *	2.30E-02	-0.201 *	2.40E-03	2.188	3.50E-08	-	-
P39059	COL15A1	-0.144 *	1.70E-02	-0.015	9.00E-01	1.865	2.70E-06	-	-
P50552	VASP	0.202 *	4.50E-04	-0.293 *	9.20E-06	2.09	3.00E-08	1.2	3.30E-02
Q9BXR6	CFHR5	0.148 *	1.60E-02	-0.055	5.50E-01	1.201	2.70E-03	1.2	7.00E-03
Q53T59	HS1BP3	-0.06	4.50E-01	-0.168 *	1.70E-02	1.497	1.70E-04	-	-
P04626	ERBB2	-0.024	8.20E-01	-0.170 *	1.70E-02	4.753	1.90E-37	-	-
Q16773	KYAT1	0.003	9.80E-01	-0.171 *	1.70E-02	2.284	1.30E-09	-	-
O43827	ANGPTL7	-0.04	6.60E-01	-0.167 *	1.70E-02	2.389	9.50E-10	-	-
Q13541	EIF4EBP1	-0.143 *	1.70E-02	-0.099	2.00E-01	1.853	3.00E-06	-	-

P63313	TMSB10	-0.132 *	2.60E-02	0.074	3.50E-01	1.685	6.70E-05	1.3	2.60E-04
Q8TCZ2	CD99L2	-0.116	7.80E-02	-0.169 *	1.70E-02	4.11	4.00E-27	1.2	9.30E-03
O95866	MPIG6B	0.244 *	4.10E-07	-0.615 *	3.80E-29	3.405	2.20E-16	1.2	3.50E-02
P02771	AFP	0.028	7.80E-01	-0.197 *	4.40E-03	1.205	2.50E-03	0.8	2.00E-02
P09467	FBP1	-0.072	3.50E-01	0.194 *	5.00E-03	0.993	1.30E-02	-	-
Q6P5S2	LEG1	0.171 *	4.70E-03	-0.177 *	1.30E-02	1.019	9.00E-03	-	-
Q6PL24	TMED8	0.181 *	2.20E-03	-0.393 *	8.90E-10	1.822	1.40E-06	1.2	3.40E-02
O75054	IGSF3	-0.059	4.60E-01	-0.166 *	1.80E-02	3.322	8.30E-18	-	-
Q9NZD4	AHSP	-0.137 *	1.80E-02	0.015	8.80E-01	1.661	4.90E-05	-	-
Q13451	FKBP5	0.136 *	3.10E-02	-0.272 *	4.30E-05	1.881	1.10E-06	1.2	5.10E-03
Q5SW79	CEP170	-0.031	7.60E-01	-0.207 *	3.00E-03	0.966	1.50E-02	-	-
P02776	PF4	-0.052	5.10E-01	-0.311 *	2.60E-07	1.054	1.30E-02	1.2	1.50E-02
Q99674	CGREF1	-0.139 *	2.60E-02	-0.209 *	2.00E-03	1.78	6.30E-06	-	-
P04083	ANXA1	0.025	8.00E-01	0.203 *	2.90E-03	0.975	1.60E-02	-	-
Q9UBC9	SPRR3	-0.168 *	3.00E-03	0.065	4.40E-01	1.013	1.60E-02	-	-
O60447	EVI5	0.139 *	2.80E-02	-0.402 *	4.70E-10	1.955	2.50E-07	-	-
Q16627	CCL14	-0.096	1.50E-01	0.163 *	1.90E-02	-	-	1.2	2.60E-04
P80188	LCN2	-0.144 *	1.80E-02	-0.058	5.20E-01	1.422	4.00E-04	-	-
Q07817	BCL2L1	0.140 *	2.80E-02	-0.261 *	9.90E-05	1.923	3.20E-07	-	-
Q5VIR6	VPS53	0.138 *	2.80E-02	-0.297 *	6.30E-06	1.493	1.00E-04	-	-
Q14210	LY6D	-0.266 *	3.80E-08	0.628 *	2.60E-29	-1.547	3.60E-04	1.2	3.80E-02
Q8N1Q1	CA13	0.259 *	4.20E-07	-0.488 *	9.40E-17	2.318	6.00E-09	1.2	3.90E-02
Q9NPH3	IL1RAP	0.038	6.80E-01	-0.355 *	1.90E-08	-	-	1.2	2.00E-02
O43805	SSNA1	0.032	7.40E-01	-0.167 *	2.00E-02	1.641	1.40E-05	-	-
P54652	HSPA2	-0.175 *	3.60E-03	-0.170 *	1.70E-02	1.029	8.80E-03	-	-
Q14643	ITPR1	0.036	7.10E-01	-0.180 *	1.20E-02	1.041	8.20E-03	-	-
Q7Z5R6	APBB1IP	-0.028	7.80E-01	0.164 *	2.00E-02	4.232	1.30E-28	-	-
Q14108	SCARB2	-0.159 *	4.20E-03	0.142 *	3.60E-02	1.567	4.30E-04	1.4	1.50E-06
P07451	CA3	-0.248 *	2.90E-06	-0.150 *	3.00E-02	1.433	4.60E-04	-	-
Q2MKA7	RSPO1	-0.087	1.70E-01	-0.157 *	1.30E-02	-	-	1.2	7.80E-03

Q76LX8	ADAMTS13	0.229 *	1.00E-05	-0.328 *	3.70E-08	2.89	7.40E-13	0.8	4.10E-02
Q9GZZ8	LACRT	0.297 *	8.20E-08	-0.086	3.10E-01	-	-	0.8	2.10E-02
P30040	ERP29	0.059	4.80E-01	-0.272 *	4.30E-05	1.579	3.60E-05	1.2	3.10E-02
P08590	MYL3	-0.340 *	2.10E-11	-0.152 *	2.70E-02	1.195	4.10E-03	-	-
P16278	GLB1	0.029	7.60E-01	-0.160 *	2.10E-02	2.925	7.80E-14	-	-
P19525	EIF2AK2	0.137 *	3.10E-02	-0.335 *	3.20E-07	1.656	1.10E-05	-	-
Q9GZM7	TINAGL1	0.005	9.60E-01	-0.164 *	1.20E-02	2.921	1.90E-12	1.2	2.00E-02
Q99616	CCL13	0.137 *	1.90E-02	-0.282 *	4.50E-06	2.476	1.30E-09	1.2	2.40E-02
O94856	NFASC	0.024	8.20E-01	-0.159 *	2.10E-02	2.659	2.80E-11	-	-
Q9H2A7	CXCL16	0.02	8.50E-01	-0.162 *	2.10E-02	2.297	5.10E-09	-	-
P25942	CD40	-0.075	3.00E-01	-0.202 *	2.30E-03	2.197	6.80E-08	1.2	3.00E-02
Q8N9I9	DTX3	-0.128 *	2.40E-02	-0.166 *	8.50E-03	3.422	7.10E-15	-	-
Q01973	ROR1	-0.018	8.60E-01	-0.159 *	2.20E-02	1.54	1.60E-04	-	-
P49747	COMP	-0.160 *	4.90E-03	-0.213 *	9.20E-04	1.347	1.20E-03	0.8	3.70E-02
Q9UMF0	ICAM5	0.017	8.80E-01	-0.172 *	1.60E-02	-	-	1.2	6.40E-03
P28325	CST5	-0.07	3.60E-01	-0.183 *	6.90E-03	-	-	1.2	1.60E-02
P23582	NPPC	-0.138 *	1.70E-02	-0.015	8.80E-01	1.186	5.60E-03	-	-
Q99942	RNF5	0.136 *	3.20E-02	-0.333 *	3.30E-07	1.214	1.80E-03	-	-
P15121	AKR1B1	0.041	6.60E-01	-0.176 *	1.50E-02	1.05	7.60E-03	-	-
P09871	C1S	0.09	2.20E-01	-0.210 *	3.00E-03	2.546	7.40E-12	1.1	3.10E-02
Q9NQ48	LZTFL1	0.136 *	3.20E-02	-0.212 *	2.00E-03	1.653	1.30E-05	-	-
P46109	CRKL	0.134 *	3.40E-02	-0.382 *	1.70E-09	2.701	9.80E-13	-	-
Q9GZX6	IL22	-0.299 *	3.40E-08	0.443 *	2.10E-12	-1.472	1.10E-04	1.2	4.60E-02
Q7Z7K0	CMC1	0.142 *	2.40E-02	-0.335 *	2.30E-07	1.002	1.10E-02	-	-
P20807	CAPN3	-0.146 *	2.30E-02	-0.182 *	1.20E-02	1.341	3.90E-04	-	-
P02458	COL2A1	-0.068	3.80E-01	-0.450 *	5.30E-13	0.915	2.30E-02	-	-
P22079	LPO	0.144 *	2.40E-02	-0.124	1.10E-01	1.48	9.90E-05	-	-
Q6NW40	RGMB	-0.066	3.80E-01	-0.157 *	2.30E-02	1.386	7.90E-04	-	-
Q6XQN6	NAPRT	-0.077	3.10E-01	0.191 *	6.30E-03	0.943	1.80E-02	-	-
P27169	PON1	0.128 *	4.50E-02	-0.394 *	4.20E-10	1.253	1.40E-03	0.8	1.40E-03

Q9UHI8	ADAMTS1	0.043	6.40E-01	-0.166 *	2.20E-02	1.19	2.50E-03	-	-
Q15303	ERBB4	0.079	2.60E-01	-0.155 *	2.30E-02	1.292	1.70E-03	-	-
Q53FA7	TP53I3	-0.142 *	2.50E-02	-0.027	8.00E-01	2.474	6.30E-11	-	-
P08571	CD14	0.138 *	2.50E-02	-0.055	5.40E-01	2.002	3.90E-07	-	-
Q96ID5	IGSF21	-0.094	1.90E-01	-0.272 *	4.00E-05	0.905	2.50E-02	-	-
P13224	GP1BB	0.129 *	3.70E-02	-0.372 *	2.40E-09	1.744	8.00E-06	-	-
Q8NI22	MCFD2	0.007	9.50E-01	-0.179 *	9.40E-03	-	-	1.2	1.50E-02
Q7Z434	MAVS	0.082	2.80E-01	-0.221 *	1.60E-03	2.077	5.50E-08	1.2	3.60E-02
Q9BSW2	CRACR2A	0.145 *	1.90E-02	-0.167 *	1.90E-02	1.815	3.10E-06	-	-
Q14696	MESD	0.117	7.30E-02	-0.322 *	5.90E-07	1.697	1.30E-05	1.2	3.80E-02
Q6P2H3	CEP85	0.107	1.20E-01	-0.164 *	2.50E-02	1.424	2.20E-04	-	-
Q03252	LMNB2	-0.130 *	3.70E-02	0.248 *	1.30E-04	1.378	6.50E-04	-	-
Q7L266	ASRGL1	0.134 *	3.80E-02	-0.293 *	9.70E-06	2.494	2.20E-11	-	-
P09960	LTA4H	-0.035	6.70E-01	0.614 *	8.00E-28	-	-	1.2	2.50E-02
P09683	SCT	0.011	9.30E-01	-0.211 *	2.00E-03	0.911	2.40E-02	-	-
P31146	CORO1A	0.130 *	3.90E-02	-0.311 *	1.30E-06	3.433	8.10E-20	-	-
P51693	APLP1	0.140 *	1.20E-02	0.063	4.30E-01	-1.722	4.90E-05	0.8	2.60E-02
Q99426	TBCB	0.173 *	3.10E-03	-0.427 *	7.20E-12	2.488	1.00E-10	1.2	4.90E-02
Q86SR1	GALNT10	0.067	3.90E-01	-0.159 *	2.60E-02	2.777	4.80E-13	-	-
P61218	POLR2F	-0.224 *	3.60E-05	0.149 *	3.40E-02	1.162	5.60E-03	-	-
Q08378	GOLGA3	0.04	6.70E-01	-0.219 *	1.60E-03	0.876	2.50E-02	-	-
P13501	CCL5	-0.083	2.00E-01	-0.357 *	6.40E-10	0.922	4.00E-02	1.4	4.00E-07
Q9P126	CLEC1B	0.123 *	4.20E-02	-0.410 *	7.70E-12	3.18	9.30E-16	1.2	1.20E-02
P15514	AREG	-0.137 *	2.60E-02	0.006	9.50E-01	-	-	1.3	1.60E-03
Q9C0C4	SEMA4C	0.136 *	1.80E-02	0.069	3.80E-01	1.113	9.80E-03	-	-
P31431	SDC4	0.112 *	4.10E-02	-0.251 *	8.20E-06	1.781	1.40E-05	-	-
P13232	IL7	0.016	8.80E-01	-0.422 *	4.10E-11	0.831	4.00E-02	1.2	1.40E-03
P17301	ITGA2	-0.149 *	1.40E-02	-0.227 *	6.20E-04	0.904	2.70E-02	-	-
Q13145	BAMBI	-0.153 *	1.10E-02	0.049	6.00E-01	-	-	1.2	1.70E-02
Q676U5	ATG16L1	0.131 *	4.20E-02	-0.371 *	8.30E-09	2.291	9.20E-10	-	-

P40189	IL6ST	-0.026	8.00E-01	-0.169 *	1.90E-02	3.26	5.90E-18	1.2	2.30E-02
Q92765	FRZB	0.138 *	2.80E-02	-0.174 *	1.40E-02	2.87	2.80E-14	-	-
P40197	GP5	-0.133 *	2.80E-02	-0.021	8.40E-01	1.509	1.00E-04	-	-
Q15517	CDSN	-0.109	8.40E-02	0.779 *	6.70E-40	-0.928	2.60E-02	1.2	1.70E-02
P48304	REG1B	-0.244 *	7.40E-06	0.176 *	1.10E-02	-0.87	3.80E-02	1.2	9.00E-03
Q96FE7	PIK3IP1	-0.156 *	5.40E-03	0.011	9.20E-01	-	-	1.2	2.30E-02
P42575	CASP2	0.072	3.60E-01	-0.170 *	1.90E-02	1.011	9.60E-03	-	-
Q8N5J2	MINDY1	0.152 *	1.30E-02	-0.414 *	5.90E-11	0.872	3.00E-02	-	-
P09417	QDPR	-0.068	3.80E-01	-0.155 *	2.90E-02	2.771	4.40E-13	-	-
Q6QNK2	ADGRD1	-0.023	8.20E-01	-0.148 *	2.90E-02	3.376	1.20E-16	-	-
P28799	GRN	-0.019	8.60E-01	0.157 *	2.90E-02	1.824	2.40E-06	-	-
Q6FHJ7	SFRP4	-0.114	8.40E-02	-0.359 *	1.90E-08	2.026	1.30E-07	0.9	4.40E-02
Q9Y5A7	NUB1	0.064	4.30E-01	-0.183 *	1.10E-02	0.934	1.80E-02	-	-
P43487	RANBP1	-0.132 *	3.50E-02	-0.185 *	7.00E-03	1.253	1.60E-03	-	-
Q9BVM4	GGACT	-0.018	8.70E-01	-0.151 *	4.10E-02	1.293	7.60E-04	1.2	3.30E-03
P06756	ITGAV	-0.081	2.50E-01	-0.230 *	4.40E-04	0.913	3.00E-02	-	-
P13807	GYS1	-0.166 *	5.40E-03	-0.150 *	4.00E-02	1.382	4.20E-04	-	-
P50591	TNFSF10	0.128 *	4.60E-02	-0.321 *	6.00E-07	2.829	4.40E-14	-	-
Q13361	MFAP5	-0.126 *	4.60E-02	-0.289 *	6.20E-06	3.294	3.60E-17	-	-
P16455	MGMT	0.129 *	4.60E-02	-0.349 *	6.90E-08	2.301	9.60E-10	-	-
P02144	MB	-0.206 *	5.60E-05	-0.212 *	5.30E-04	0.902	4.50E-02	-	-
Q8TAT2	FGFBP3	0.023	8.20E-01	-0.254 *	6.40E-05	0.96	2.50E-02	1.2	2.10E-02
Q9HD26	GOPC	0.133 *	3.60E-02	-0.390 *	1.10E-09	2.03	9.20E-08	1.2	2.60E-02
Q8WYQ3	CHCHD10	-0.127 *	3.00E-02	-0.017	8.70E-01	1.408	1.30E-03	-	-
O43521-2	BCL2L11	-0.079	2.90E-01	-0.314 *	1.40E-06	-	-	1.2	3.20E-02
P08962	CD63	-0.123 *	4.70E-02	-0.210 *	1.30E-03	1.743	1.80E-05	-	-
P48960	ADGRE5	0.121 *	4.90E-02	-0.360 *	3.60E-09	3.79	3.00E-22	-	-
Q9H4X1	RGCC	0.149 *	1.40E-02	-0.251 *	1.40E-04	0.843	3.40E-02	-	-
Q8IXS6	PALM2	-0.134 *	3.30E-02	0.075	3.70E-01	2.952	2.60E-14	-	-
Q00872	MYBPC1	-0.352 *	2.60E-12	-0.138 *	4.60E-02	1.23	2.80E-03	-	-

Q6UVK1	CSPG4	-0.089	1.60E-01	-0.142 *	3.10E-02	1.401	1.40E-03	-	-
Q9Y4K4	MAP4K5	0.127 *	5.00E-02	-0.393 *	1.20E-09	2.21	6.00E-09	-	-
Q04637	EIF4G1	0.081	2.80E-01	-0.209 *	2.70E-03	2.82	6.10E-14	1.2	4.80E-02
P40121	CAPG	-0.143 *	1.30E-02	0.083	2.80E-01	1.15	8.90E-03	1.2	2.90E-02
P01135	TGFA	-0.190 *	6.00E-04	0.045	6.20E-01	-	-	1.2	3.30E-02
O77932	DXO	0.067	4.00E-01	-0.166 *	2.20E-02	0.981	1.20E-02	-	-
P07148	FABP1	-0.162 *	4.20E-03	-0.027	7.80E-01	1.43	5.90E-04	1.2	4.60E-02
Q96NY8	NECTIN4	-0.084	2.20E-01	0.244 *	1.10E-04	-0.927	3.40E-02	-	-
Q6UW56	ATRAID	-0.142 *	1.70E-02	-0.023	8.20E-01	1.014	1.70E-02	-	-
P17538	CTRB1	0.118	7.10E-02	-0.258 *	9.40E-05	0.861	3.40E-02	-	-
P13521	SCG2	-0.079	2.20E-01	-0.205 *	7.30E-04	-	-	1.2	3.40E-02
P20701	ITGAL	-0.159 *	6.90E-03	0.264 *	4.30E-05	0.823	4.70E-02	-	-
P98082	DAB2	0.137 *	2.80E-02	-0.213 *	1.50E-03	2.274	4.40E-09	1.2	4.40E-02
O43557	TNFSF14	-0.132 *	3.70E-02	-0.013	9.10E-01	2	2.60E-07	-	-
P23560	BDNF	-0.069	3.30E-01	-0.362 *	6.30E-10	0.91	3.70E-02	1.2	1.80E-02
P01210	PENK	-0.129 *	3.60E-02	-0.117	1.10E-01	1.29	1.90E-03	-	-
P55789	GFER	-0.139 *	2.90E-02	-0.159 *	2.80E-02	1.323	8.40E-04	-	-
Q9UFP1	GASK1A	-0.134 *	3.90E-02	-0.074	4.00E-01	2.58	4.70E-12	-	-
P80075	CCL8	0.132 *	3.90E-02	-0.240 *	3.40E-04	1.2	2.30E-03	1.2	3.60E-02
P27487	DPP4	-0.165 *	4.90E-03	0.023	8.30E-01	0.874	3.40E-02	-	-
P21980	TGM2	-0.125 *	3.90E-02	-0.001	9.90E-01	1.407	5.40E-04	-	-
Q8IXQ3	C9orf40	-0.146 *	1.40E-02	0.037	7.00E-01	0.918	2.50E-02	-	-
Q9UJ68	MSRA	0.126	5.50E-02	-0.166 *	2.10E-02	3.416	5.10E-20	1.2	3.80E-02
Q9Y3D6	FIS1	-0.06	4.50E-01	-0.149 *	3.90E-02	2.392	5.30E-10	-	-
P04275	VWF	0.159 *	7.50E-03	-0.145 *	4.60E-02	1.173	3.50E-03	1.2	2.20E-02
Q9NPH6	OBP2B	-0.042	6.30E-01	0.415 *	4.90E-12	-	-	1.2	4.00E-02
P52630	STAT2	0.127	5.20E-02	-0.151 *	3.90E-02	1.236	1.70E-03	-	-
P80098	CCL7	-0.167 *	3.40E-03	0.150 *	2.90E-02	0.878	4.60E-02	1.3	2.80E-03
Q9BXI3	NT5C1A	-0.124 *	4.20E-02	-0.047	6.10E-01	3.061	7.20E-14	-	-
Q08830	FGL1	0.127 *	4.30E-02	-0.200 *	2.80E-03	-0.903	3.00E-02	1.2	7.60E-03

P98170	XIAP	0.145 *	2.10E-02	-0.150 *	4.10E-02	1.476	1.40E-04	-	-
Q9NRM6	IL17RB	0.061	3.80E-01	-0.132 *	4.30E-02	-1.949	8.40E-06	-	-
Q9H2K0	MTIF3	0.150 *	1.70E-02	-0.09	2.80E-01	0.882	2.50E-02	-	-
P07355	ANXA2	-0.131 *	4.30E-02	-0.116	1.40E-01	1.356	4.40E-04	-	-
P02760	AMBP	-0.004	9.80E-01	-0.153 *	2.60E-02	4.484	7.90E-28	1.2	3.90E-02
Q9NZV1	CRIM1	-0.04	6.20E-01	-0.132 *	4.20E-02	1.395	2.50E-03	-	-
O43927	CXCL13	-0.157 *	9.60E-03	0.150 *	3.90E-02	0.949	1.80E-02	-	-
Q16363	LAMA4	-0.129 *	3.80E-02	-0.393 *	2.40E-10	1.128	5.70E-03	1.2	4.60E-02
Q13442	PDAP1	0.062	4.60E-01	-0.198 *	5.10E-03	-	-	1.2	4.00E-02
P15502	ELN	-0.144 *	1.50E-03	-0.009	9.20E-01	1.176	4.20E-02	1.3	2.50E-02
P00918	CA2	-0.121 *	4.40E-02	0.049	5.80E-01	1.284	1.90E-03	-	-
P24666	ACP1	-0.157 *	1.10E-02	0.105	1.90E-01	-	-	1.2	3.70E-02
Q3KPI0	CEACAM21	-0.153 *	1.30E-02	-0.057	5.30E-01	0.833	4.30E-02	1.2	1.70E-02
Q96PQ0	SORCS2	-0.236 *	8.40E-06	-0.139 *	4.70E-02	-	-	1.2	2.60E-02
Q86VB7	CD163	-0.156 *	2.20E-03	0.01	9.20E-01	0.963	4.70E-02	-	-
Q9Y266	NUDC	0.058	4.90E-01	-0.147 *	5.00E-02	2.847	2.00E-14	1.2	2.50E-02
Q8N2Q7	NLGN1	-0.02	8.50E-01	-0.163 *	2.70E-02	-	-	1.2	2.40E-02
P26447	S100A4	-0.121	6.70E-02	0.190 *	6.50E-03	0.808	4.50E-02	-	-
Q99972	MYOC	0.026	7.90E-01	-0.147 *	3.90E-02	1.031	1.30E-02	-	-
P01138	NGF	0.011	9.30E-01	-0.195 *	6.50E-03	-	-	1.2	4.70E-02
Q12841	FSTL1	-0.159 *	8.40E-03	-0.115	1.40E-01	0.842	4.60E-02	-	-
Q08629	SPOCK1	0.093	1.90E-01	-0.149 *	4.00E-02	0.993	1.50E-02	-	-
P29317	EPHA2	-0.170 *	2.10E-03	0.001	1.00E+00	0.926	4.50E-02	1.2	3.60E-02
Q9UI42	CPA4	0.068	3.90E-01	-0.164 *	2.40E-02	0.846	3.30E-02	-	-
Q16740	CLPP	0.037	7.00E-01	-0.176 *	1.50E-02	0.807	4.50E-02	-	-
Q8N386	LRRC25	-0.147 *	1.70E-02	0.089	2.80E-01	-	-	1.2	4.40E-02
P02647	APOA1	0.063	4.30E-01	-0.159 *	2.70E-02	0.841	3.70E-02	-	-
Q8NHL6	LILRB1	-0.123 *	4.60E-02	0.029	7.70E-01	0.987	1.90E-02	0.9	3.60E-02

Abbreviation: SBP, systolic blood pressure; IHD, ischemic heart disease; NPX, Normalized Protein eXpression; HR, hazard ratio; FDR, false discovery rate; BMI, body mass index.

* FDR P value < .05 (for temperature-related proteins only).

[a] Models are adjusted for relative humidity, region, year of sample collection, fasting time, fasting time^2, age, age^2, sex, hour of blood collection, hours to blood processing, and case ascertainment status.

[b] Models are adjusted for region, fasting time, fasting time^2, age, age^2, sex, and plate ID.

[c] Models are adjusted for region, fasting time, fasting time^2, age, age^2, sex, education, smoking, alcohol, total physical activity, SBP, diabetes, BMI and plate ID.

[d] Changes in NPX at 5th percentile (-2.1 °C) v.s. median (17.7 °C) temperature.

[e] Changes in NPX at 95th percentile (29.5 °C) v.s. median (17.7 °C) temperature.

[f] Per a unit change in NPX.
